# Natural history models for lung Cancer: A scoping review

**DOI:** 10.1016/j.lungcan.2025.108495

**Published:** 2025-03-26

**Authors:** Renu Sara Nargund, Sayaka Ishizawa, Maryam Eghbalizarch, Paul Yeh, Seyyed Mostafa Mousavi Janbeh Saray, Sara Nofal, Yimin Geng, Pianpian Cao, Edwin J. Ostrin, Rafael Meza, Martin C. Tammemägi, Robert J. Volk, Maria A. Lopez-Olivo, Iakovos Toumazis

**Affiliations:** a Department of Health Services Research, University of Texas MD Anderson Cancer Center, Houston, TX, USA; b Department of Management, Policy, and Community Health, University of Texas Health Science Center at Houston School of Public Health, Houston, TX, USA; c Research Medical Library, University of Texas MD Anderson Cancer Center, Houston, TX, USA; d Department of Public Health, Purdue University, West Lafayette, IN, USA; e General Internal Medicine, University of Texas MD Anderson Cancer Center, Houston, TX, USA; f British Columbia Cancer Research Institute, Vancouver, British Columbia, Canada; g School of Population and Public Health, University of British Columbia, Vancouver, British Columbia, Canada; h Department of Health Sciences, Brock University, St. Catharines, Ontario, Canada

**Keywords:** Simulation modeling, Lung cancer, Natural history

## Abstract

**Introduction:**

Natural history models (NHMs) of lung cancer (LC) simulate the disease’s natural progression providing a baseline for assessing the impact of interventions. NHMs have been increasingly used to inform public health policies, highlighting their utility. The objective of this scoping review was to summarize existing LC NHMs, identify their limitations, and propose a framework for future NHM development.

**Methods:**

We searched MEDLINE, Embase, Web of Science, and IEEE Xplore from their inception to October 5, 2023, for peer-reviewed, full-length articles with an LC NHM. Model characteristics, their applications, data sources used, and limitations were extracted and narratively synthesized.

**Results:**

From 238 publications, 69 publications were included in our review, corresponding to 22 original LC NHMs and 47 model applications. The majority of the models (n = 15, 68 %) used a microsimulation approach. NHM parameters were predominately informed by cancer registries, trial and institutional data, and literature. Model quality and performance were evaluated in 8 (36 %) models. Twenty (91 %) models included at least one carcinogenesis risk factor–primarily age, sex, and smoking history. Three (14 %) LC NHMs modeled progression in never-smokers; one (5 %) addressed recurrence. Non-tobacco smoking, nodule type, and biomarker expression were not considered in existing NHMs. Based on our findings, we proposed a framework for future LC NHM development which incorporates recurrence, nodule type differentiation, biomarker expression levels, biological factors, and non-smoking-related risk factors.

**Conclusion:**

Regular updating and future research are warranted to address limitations in existing NHMs thereby ensuring relevance and accuracy of modeling approaches in the evolving LC landscape.

## 1. Introduction

Natural history models (NHMs) of lung cancer (LC) are crucial as they provide a baseline understanding of the development and progression of LC in the absence of interventions. Simulating the natural LC progression provides the opportunity to assess, in silico, the benefits and harms associated with potential interventions. Furthermore, NHMs can be used to evaluate counterfactual scenarios, such as the impact of various levels of screening uptake or eradication of specific LC risk factors in the population, thus providing important insights to decision-makers. Therefore, NHMs play an important role in shaping public health policies and clinical strategies, ultimately aiming to improve patient outcomes.

Historically, the development of LC NHMs has been influenced by key studies and advances in the field. Geddes conducted a pivotal study that initially mapped out the growth rates of untreated lung tumors, providing a foundational dataset used to benchmark newer models.[1] The availability of more comprehensive lung tumor data and technological advancements in imaging improved our understanding of the disease progression and its preclinical phases. The establishment of randomized controlled trial datasets from major screening trials like the Mayo Lung Project[2,3], Mayo CT Screening Study[4], Early Lung Cancer Action Project (ELCAP)[5,6], Prostate, Lung, Colorectal, and Ovarian (PLCO) Cancer Screening Trial[7], National Lung Screening Trial[8] (NLST), and the Dutch-Belgian Randomized Lung Cancer Screening Trial (NELSON),[9] along with advancements in computational power, has led to more complex models, enhancing their accuracy and applicability.[10].

LC NHMs have been increasingly utilized to inform public health policy. The Cancer Intervention and Surveillance Modeling Network [11] (CISNET) consortium in the US conducted comparative modeling analyses using independently developed NHMs to inform, among others, the US Preventive Services Task Force recommendations on lung cancer screening.[12,13] Moreover, LC NHMs have been extensively applied across various contexts to assess the cost-effectiveness of several interventions, mainly for secondary prevention and early detection. [14–16].

The evolving LC landscape necessitates frequent update of these models to ensure they reflect the current state of LC development and progression. Historically, the majority of LC cases were attributed to smoking; however, LC in never-smokers is now the fifth leading cause of cancer-related death worldwide.[17] Additionally, the impact of e-cigarettes and other forms of non-combustible smoking on LC incidence and mortality rates remains unknown. The influence of other exogenous risk factors, such as air pollution and occupational exposure, will inevitably continue to rise as tobacco smoking prevalence declines.[18] Calibration, external validation, and sensitivity analyses are crucial practices following model development to ensure relevance and generalizability of the models to populations not used during model development.[19] Advances in lung cancer screening modalities, such as the shift from chest X-rays to low-dose CT, could offer greater insights into the preclinical phase of the disease that may warrant updating existing models.

This is the first scoping review undertaken of LC NHMs, which aims to catalog and summarize existing LC NHMs and identify opportunities for future research and NHM development.

## 2. Methods

The scoping review was based on an existing protocol (https://doi.org/10.17605/OSF.IO/MSP4Q) submitted to the Open Science Framework (OSF) and is reported in accordance with the PRISMA Extension for Scoping Reviews (Supplementary Table S1).[20].

### 2.1. Eligibility criteria

We included studies that described and used models of LC development and progression from its onset to death in the absence of any intervention (i.e., screening or treatment). Specifically, the models had to satisfy the criteria of a NHM[21]; that is, to capture the various stages and transitions within the progression of LC itself, including tumor development, growth, progression from presymptomatic phase to clinical disease and from localized to advanced stages, and survival. Articles were excluded if a LC forecasting model was not introduced or described (ex. genomic association or retrospective case analysis). Next, exclusion was also upheld for models: 1) without a natural history component (ex. model lung cancer only in the presence of screening or treatment interventions); 2) that focused on a sub-component of LC progression (ex. incidence, survival); or 3) without sufficient detail on LC progression (ex. no stage-progression or a 1-year time horizon). We also excluded articles not written in English language, not peer-reviewed, not published as full-text (i.e., preprints, conference or meeting abstracts), and editorials.

### 2.2. Search strategy and selection criteria

A librarian expert in literature reviews (YG) searched MEDLINE (through Ovid), Embase (through Ovid), Web of Science (through Clarivate), and IEEE Xplore databases from their inception through October 5, 2023. The terms included in the search strategy were grouped into three categories: 1) LC; 2) natural history; and 3) modeling, with relevant terms from each category combined to yield the final search set. The final search strategy was modified as needed for each database (Supplementary Table S2) to account for differences in keywords, MeSH terms and syntax across databases. All retrieved results were consolidated in EndNote 21[22] (Clarivate; London, UK) and duplicates were removed. The references of the included studies and relevant reviews[14,23] were manually searched for additional publications meeting our inclusion criteria.

### 2.3. Selection and screening process

The selection process for sources of evidence involved a two-step approach using Covidence.[24] The title and abstract of each reference were independently reviewed by pairs of reviewers (RSN, SI, PY, and SMJS) to identify potentially relevant citations. Citations that passed the title/abstract review were retrieved for full-text review and were further screened (RSN, PY, SI, ME, SN, and SMJS). Disagreements at both stages were resolved through discussion or by a third author (IT) if consensus among the reviewers was not achieved.

### 2.4. Data extraction and summary

Data extraction was performed independently by pairs of reviewers to ensure accuracy (RSN, SI, and ME) using a standardized Excel form, which was pilot-tested and iteratively developed by the team to ensure it comprehensively captured all relevant data. Any disagreements during this process were resolved through discussion or by consulting a third reviewer (IT).

### 2.5. Data items

We collected general study characteristics (authors, study year, country, and intended use of the NHM), data used to inform model’s input parameters (incidence, prevalence, mortality, stage and histology distributions, and transition probabilities), variables influencing key elements of the models (carcinogenesis and risk factors, cancer progression, staging, histological types, use of a risk model), model validity (calibration, validation, sensitivity analysis, limitations, and proposed extensions), and model evolution over time (changes to model structure, inclusion of additional risk factors, number of applications).

### 2.6. Synthesis of results

Descriptive statistics were used to summarize the data on all variables collected across three model domains defined based on the NHM criteria. The incidence domain captures lung cancer onset or tumor development. Second, the natural history domain is the pre-clinical progression phase. Third, the recurrence natural history domain covers the onset and progression of recurrent LC malignancies. We used a tabular approach to present our results across the three domains. Different iterations and applications of the same model were identified using the model’s name and based on references to previous papers that describe the development of the model. Novel applications of a previously described model (e.g., new populations, new interventions) or recalibration and validation using new datasets were catalogued as updates. Major changes in the structure or assumptions of previously developed models were included as new iterations of the model. We conducted a qualitative synthesis of the limitations discussed by the authors of the papers to inform the development of a development framework for future LC NHMs.

## 3. Results

**Fig. 1.**
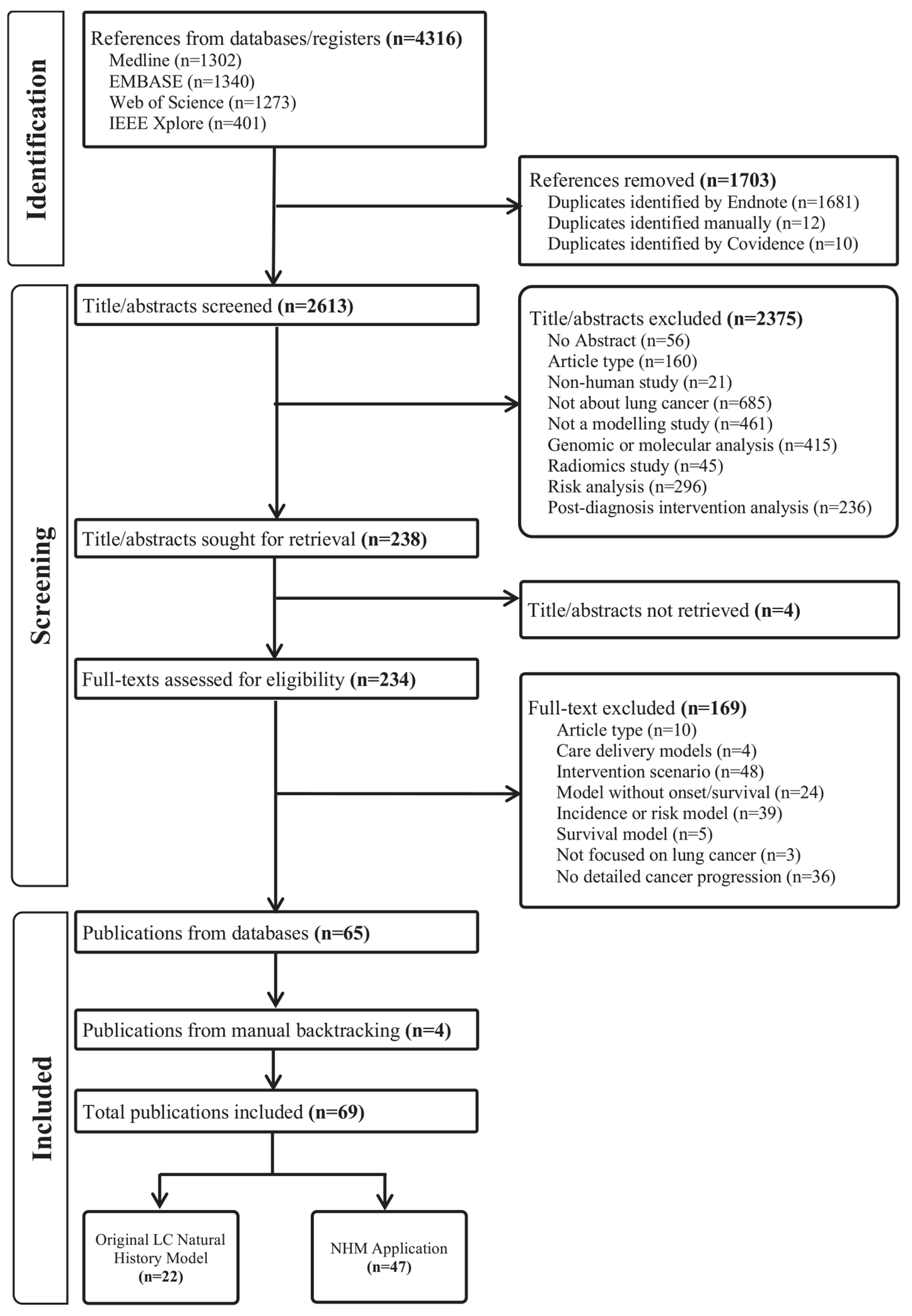
PRISMA Diagram.

Our database search yielded 2,613 unique records, of which, 234 records were identified as relevant for full-text review. A final set of 69 publications[1,12,13,15,16,25–88] were included in our review accounting for 22 unique LC NHMs[1,25–27,31,34,38,43,45,47,48,52,54,60,63,65,71,74,80,81,87,88] and 47 applications or updates of these models (Fig. 1).[12,13,15,16,28–30,32,33,35–37,39–42,44,46,49–51,53,55–59,61,62,64,66–70,72,73,75–79,82–86] A full list of excluded articles is available in Supplementary Appendix A.

Among the 22 unique models, eight were used in at least two publications,[27,31,34,37,38,43,47,74]. If multiple references are applicable with a model, we default to citing the most recent publication when referring to that model. To facilitate readability, a summary of the LC NHMs (Table 1) and a more detailed profile of each model (Supplementary Table S3) are provided.

Of the 22 models included in our search (Table 1), most (n=15, 68%) were microsimulation models, followed by Markov cohort models (n=4, 18%), tumor growth models (n=2, 9%), and decision trees (n=1, 5%).

Age, smoking history, and sex were the most common carcinogenesis risk factors captured in the incidence domain of the models. Ten (45%) models included all three risk factors,[43,45,47,54,56,62,65,67,71,88] whereas two (9%) models did not include any[52,81]. Among the models that included smoking history as a risk factor, nine (41%) further subcategorized it by smoking status, duration, intensity, and years since cessation, whereas the remaining models included a subset of smoking history factors, of which four (18%) only included smoking status, one (5%) included smoking status and years since cessation, one (5%) included smoking status and duration, one (5%) included smoking status, duration, and intensity, and one (5%) did not provide any information regarding further subcategorization of smoking history. Six models (27%) included never-smoking individuals in their LC risk assessment. Additional carcinogenesis risk factors considered by existing LC NHMs included chronic obstructive pulmonary disease (COPD) (n=2, 9%), Human immunodeficiency virus (HIV) (n=1, 5%), family and personal history of cancer (n=1, 5%), and body mass index (BMI) (n=1, 5%). Ten (45%) NHMs (all of which were microsimulation models) explicitly modeled the risk of lung carcinogenesis using a multistage carcinogenesis model.

The level of detail used to simulate LC progression varied significantly across the models. Ten (45%) NHMs simulated the progression of LC by explicitly modeling the tumor growth. Among these, six (27%) assumed an exponential tumor growth, three (14%) assumed Gompertzian growth, and one (5%) used both. Six (27%) models assumed that tumor growth rates follow a log-normal distribution, and one (5%) model used a gamma distribution. We were unable to identify the growth rate distribution for three (14%) models. Twelve (55%) NHMs used state-transition probabilities to model LC progression. Three (14%) models captured unique features of LC progression for never-smoking individuals by using a varied histology distribution or survival functions for this sub-population.

Fourteen (64%) NHMs simulated disease progression based on specific histology subtypes. All of these models modeled non-small cell lung cancer (NSCLC), but only nine (41%) considered small cell lung cancer (SCLC). Four (18%) NHMs considered NSCLC as a single cancer type with no further subdivision. Ten models (45%) used specific histology classifications, such as adenocarcinoma (AD) (n=5, 23%), AD in situ (AIS) (n=3, 14%), large cell carcinoma (LCC) (n=5, 23%), and squamous cell carcinoma (SQ) (n=9, 41%), and combinations of these: two models combined AD with LCC (9%); two combined AD with AIS (9%); and one (5%) modeled a combination of AD, LCC, and AIS. Five (23%) models included other NSCLCs as a separate category.

The staging methods varied reflecting different approaches to categorizing LC progression. NSCLC was modeled using the American Joint Committee on Cancer (AJCC) staging system in seven models (32%), TNM staging in three models (14%), and Surveillance, Epidemiology, and End Results (SEER) staging (local, regional, and distant) in four models (18%). In seven models (32%) models, AJCC stages were aggregated to approximate early and advanced LC. One (5%) model mapped TNM stages to the SEER staging system.

Factors influencing LC-specific mortality was available for 20 (91%) NHMs. Stage-specific survival was incorporated into 11 models (50%). Two (9%) models stratified LC progression by histology, but did not include its influence on survival. Age (n=5, 23%) and sex (n=7, 32%) were also commonly used to stratify LC-specific survival. Two (9%) models did not include competing causes of death.

Similar data sources were used to inform the NHMs (Table 2).These sources also evolved with time and model development (Supplementary Table S4). Country-specific cancer registries were used by several models to inform LC incidence (n=8, 36%), disease-specific mortality (n=8, 36%), and distributions for LC histology and stage (n=6, 23%). Models that were developed to model tumor growth (n=2, 9%) primarily used literature to inform tumor size and growth rate distributions. Models developed after 2008 began retrieving tumor size information from the SEER registry (n=4, 18%). Other sources for LC incidence included trial data, carcinogenesis models, and literature. Only one (5%) model did not report using any data for incidence.[1] Two (9%) models used data on LC prevalence to supplement incidence data, whereas two (9%) models used all-cause mortality estimates to derive cause-specific mortality rates. Six (27%) models relied on related literature, one used clinical expert opinion (5%), and three (14%) exclusively used global data sources to inform disease-specific mortality in their initial model. Shared resources from the National Cancer Institute, including a smoking history generator,[89,90] were also used across multiple models to adjust mortality for smoking history. Five (40%) models relied on life tables or national statistical agency resources to inform mortality and survival.

**Fig. 2.**
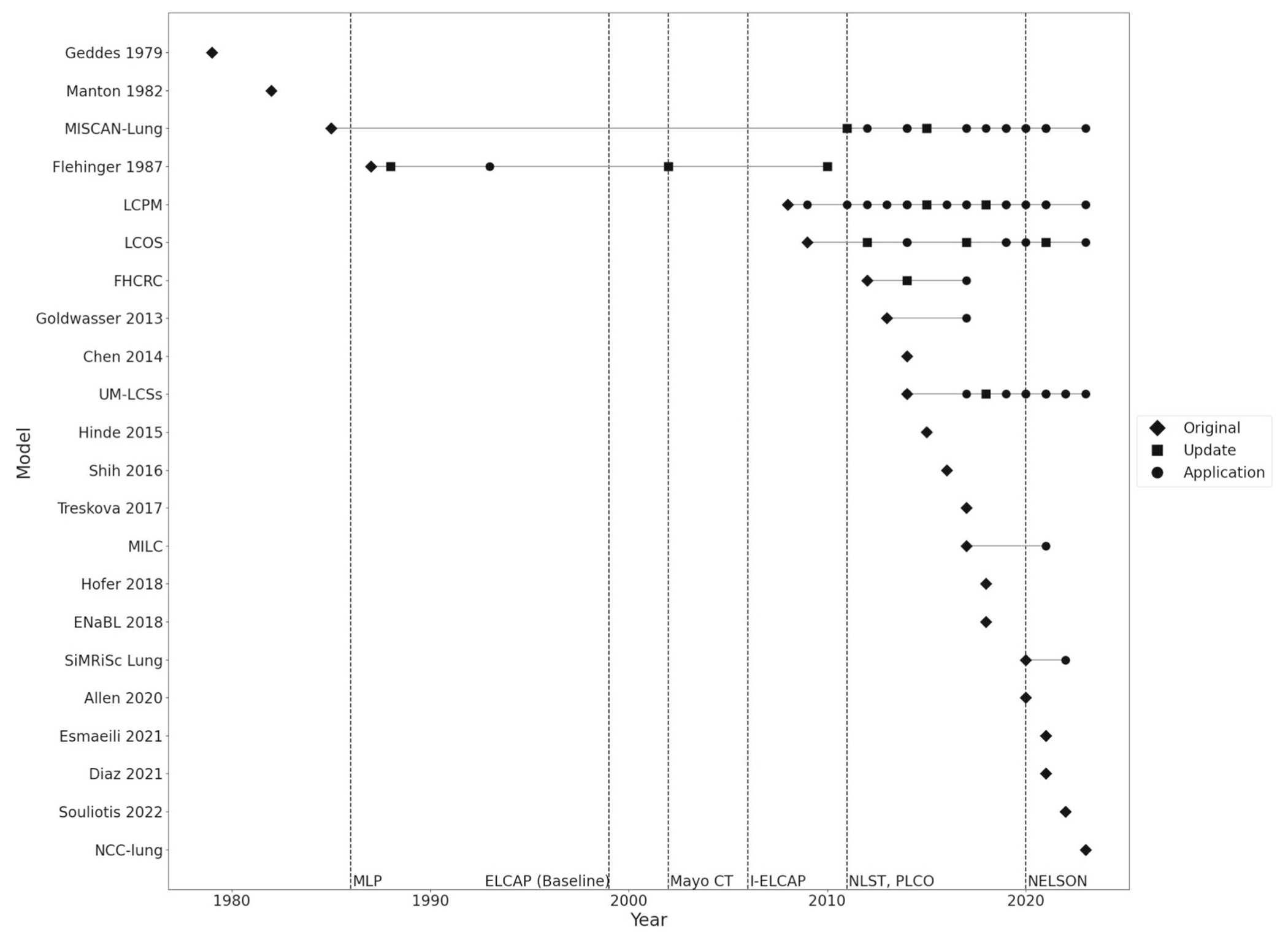
LC NHM development and application over time (n = 69) Note: Vertical dashed lines correspond to the year of publication of the corresponding study’s primary endpoint; Update indicates a change to the model’s structure; Application indicates use of the model with no structural change; In cases where models have both an update and an application in the same year, the update is shown on the figure, leading to differences between the number of articles included in the figure and the number of articles shown.

Trial data proved to be important for NHM development and subsequent updates (Fig. 2). Mayo Lung Project data was first incorporated into an LC NHM in 1993 and used by three models (14%). Mayo CT data was used once (5%) to validate LC incidence. Results published from the ELCAP were used to inform nodule growth in one model (5%). Nine models (41%) leveraged NLST data, of which five (22%) were specifically recalibrated and validated to incorporated it along with results from PLCO. The 22 models were used 104 times, including comparative analyses. The majority of instances (88%) were after 2011, the year data from NLST and PLCO was released.

Parameters of the NHMs were calibrated in 14 models (64%) [35,43,47,48,54,56,60–63,67,71,80,88] and validated in 14 (64%) models[1,25,35,43,45,47,48,54,56,60–62,67,88] (Supplementary Table S4–S5). While sensitivity analysis was performed in most articles, parameters specific to the NHM were tested in 15 models (68%). [35,43,45,47,48,52,56,60–63,65,67,80] Of the 22 models included, only eight (36%) conducted all three methods of evaluating model performance.[35,43,47,48,56,60,62,67] LC incidence (n=13, 59%) [35,43,47,48,54,56,60–63,67,80,88] and disease-specific mortality (n=10, 45%)[35,43,47,56,61–63,67,80,88] were the most commonly used calibration targets. The same holds true for validation with eight (36%)[47,54,56,60–62,67,88] models using incidence and six (27%) [1,47,56,61,67,88] models using disease-specific mortality as a validation target.

The data sources used to inform, calibrate, and validate the LC NHMs vary significantly in terms of geography (Supplementary Table S3). Twelve (54%) models were developed for and applied to the US population.[25,35,43,45,47,52,54,56,61,62,67,71] Eight (36%) models were developed for European countries: three models (14%) were developed for the United Kingdom,[1,48,65] two (9%) for Germany,[60,63] one (5%) for the Netherlands,[67] one (5%) for Greece,[87] and one (5%) for Spain.[80] Only two (9%) models were developed for applications to non-high income countries – one (5%) for China[88] and one (5%) for Iran[81]. The LC Policy and MISCAN-Lung models were both originally developed for the USA, but were adapted for applications in other countries.[58,59,67,68] Notably, our review did not identify a model developed for use in countries from Africa, South Asia, or South and Central America.

Overall, there were 47 captured studies where previously developed models were described in further detail, updated, applied. Six (27%) NHMs underwent at least one update after its initial development. [29,31,34,37,47] Five (23%) NHMs were calibrated and validated multiple times (n=5, 23%), incorporated new data, improved modeling of LC progression by expanding histological subtypes, and added carcinogenesis risk factors (n=5, 23%).[47,56,61,62,67] One (5%) model expanded their staging from an early and advanced structure[42] to the AJCC staging system,[47] and three models incorporated a carcinogenesis model[35,42,47].The most commonly used models beyond their initial publication were models from the CISNET consortium; namely, the MISCAN-Lung,[51,59,66,67,72] the Lung Cancer Policy Model,[31–33,36,41,44,49,53,58,62,64,68] the Lung Cancer Outcomes Simulator,[39,56,70,77,82,83] and the Lung Cancer Screening Model.[61,73,78,84,86] These models were also used in several comparative studies.[12,13,15,16,40,46,47,57,69,76].

Limitations of LC NHMS that were explicitly described were summarized by model domain (i.e. incidence, NHM, and recurrence NHM) (Table 3). The most common limitation for the incidence domain, noted in 10 (45%) publications, was coverage of LC risk factors. In the primary NHM domain, studies assumed that tumor growth was constant and uniformly increasing in a spherical shape (n=9, 41%). Five (22%) articles noted that their analysis only accounted for the primary nodule. One NHM[45] incorporated the growth of a primary metastatic tumor, whereas another[41] modeled the growth of both benign and malignant nodules.

Under the recurrence domain, three articles acknowledged that recurrence was not well captured in their NHM.[1,72,80] Out of the 22 LC NHMs, two models included a component related to recurrence,[1,63] but only one modeled progression of recurrent disease assuming the same growth rate as the primary tumor.[1] Only one model incorporated a biomarker but did not apply it to modeling carcinogenesis or LC progression; rather, a hypothetical biomarker was incorporated for clinical management.[83].

None of the LC NHMs included in our review stratified LC development or progression by the visual representation or attenuation of nodules. Two articles acknowledged that their model was limited to solid nodules.[32,71] It was noted that this limitation is particularly relevant when estimating the effectiveness of screening interventions.

Three articles noted severe data limitations,[63,81,87] of which, two used expert opinion to derive initial transition probabilities.[63,81] One article highlighted the need to further stratify progression of LC in NHMs by biological features that would influence the rate of cancer growth. [41] Furthermore, a dearth of regional-level data[80,81,87] and the simulation of single birth cohorts[16,67,73,76,77] was noted as a limitation in three and five articles, respectively, thus limiting the generalizability of their findings.

**Fig. 3.**
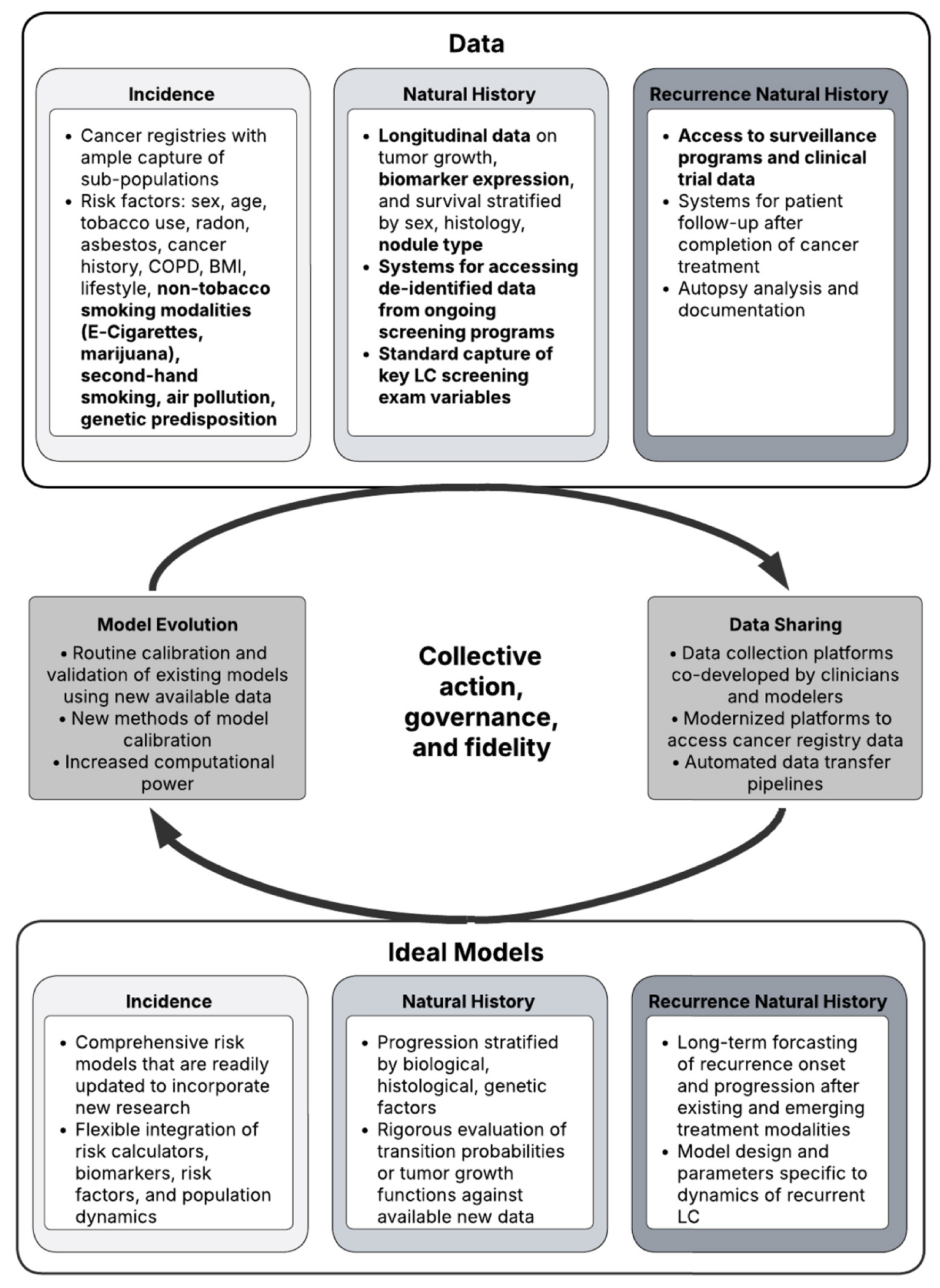
Extended Lung cancer NHM framework. *Note:* This figure presents a framework for an evolving LC NHM and data sharing system. Modelling and data collection efforts are structured by three model domains—incidence, natural history, and recurrence natural history. Collective action, governance, and fidelity sit in the middle of data sharing and model evolution systems. Data limitations identified through the current review were bolded.

We propose future steps to address existing limitations to allow for even more granular LC natural history modeling (Table 3). The implementation of these recommended steps would allow for an expanded and dynamic LC NHM framework (Fig. 3) that better captures LC natural disease progression across all model domains and facilitate consistent model evolution. Our recommendations propose the expansion of existing models through the integration of the following features: (i) non-smoking related risk-factors beyond age and sex (e.g., family history, race and ethnicity, air pollution and other environmental exposures, COPD, among others); (ii) other forms of tobacco exposure (e.g., e-cigarettes and heated-tobacco use, second hand smoking); (iii) stratification by nodule attenuation (e.g., solid, sub-solid, and ground glass opacities); (iv) biomarker expression levels; and (v) recurrence modeling. In addition to highlighting key components required to address existing limitations of NHMs (Table 3), we emphasize the data needed to consistently inform the expanded LC NHM and propose opportunities to facilitate data sharing and model evolution [91].

## 4. Discussion

In this scoping review, we collated and summarized existing LC NHMs. Key findings from this study include: 1) wide variability in methods employed to model LC progression; 2) limited geographic distribution of modeling efforts, with no models published from low- and lower-middle-income countries; 3) lack of longitudinal data to inform the models; and 4) increasing trend in complexity and improvement in precision from earlier to current models. We identified key limitations of existing models and used this information to develop a development framework for future LC NHMs.

There is an urgent need to integrate non-smoking related risk factors into existing LC NHMs. Age, sex, and smoking history remain the primary drivers of LC incidence, despite the continuous decline in smoking prevalence. Nevertheless, non-smoking-related risk factors, such as air pollution and genetic predisposition, are expected to play a more significant role in future LC.[92] In addition to impacting incidence rates, these non-smoking risk factors can also affect the natural history of LC. For example, studies have demonstrated differences in disease progression between never-smokers and ever-smokers.[92–95] Hence, future NHMs should explicitly stratify disease progression based on available non-smoking risk factors. Because future LC NHMs are likely to become more diverse as different groups progressively add additional risk factors, there is a need for establishing a comprehensive framework for future LC NHMs development.

Alternative forms of tobacco inhalation, such as e-cigarettes or heated tobacco use, are also gaining popularity, potentially altering the future LC landscape.[96] While these alternatives are marketed as healthier than traditional cigarette smoking, their long-term impact on LC incidence and mortality remains unclear, particularly considering their rising use among youth.[96–98] Given the uncertainty surrounding these trends, future LC NHMs might incorporate alternative forms of tobacco inhalation, as well as other substances such as marijuana and its derivatives, into estimations of lung carcinogenesis risk, progression, and survival.

A key opportunity for future LC NHMs is accounting for the documented variability in lung cancer progression based on pulmonary nodule classification.[99–101] Lung nodules are categorized by their attenuation as solid, part-solid, or non-solid, with each type exhibiting distinct disease progression patterns, as shown by emerging data on their respective tumor volume doubling times.[102] Clinical guidelines, such as the Lung Imaging Reporting and Data System (Lung-RADS)[103] the Fleischner Society,[104] and the British Thoracic Society,[104] recommend varying follow-up and treatment approaches based on nodule type. In our review, 45% of the LC NHMs include a tumor growth component that simulates change in tumor size over time. However, most of the data informing lung tumor growth came from limited cancer registry data and small studies.[105] Advancements in imaging technology (adoption of annual CT screening and the accumulation of longitudinal scans from patients in screening programs) and radiomic algorithms (Sybil[106] and Median[107]), provide a unique opportunity to better characterize the natural history of lung nodules by eliminating errors from human interpretation of radiological imaging[108]. Future LC NHMs should reflect heterogeneity in disease progression by nodule classification and leverage emerging data and new methods for measuring nodule size and assessing malignancy risk.

Similarly, the integration of genetic classifications into NHMs is crucial for accurately capturing LC progression and response to treatment. The discovery of immune checkpoint inhibition therapy clearly show the significant influence that genetic factors have on the progression and management of LC.[109] Several studies have documented how the expression levels of certain genes, such as epidermal growth factor receptor (EGFR) or Kirsten rat sarcoma viral oncogene homolog (KRAS), change over the course of LC.[110,111] However, these genetic factors have never been explicitly modeled in NHMs. Future models should leverage the abundances of available genetic data to improve their accuracy.

Recent advancements in screening technologies, such as multicancer early detection (MCED) tests, warrant incorporating biomarker data into NHMs. MCEDs detect circulating tumor deoxyribonucleic acid (DNA) through circulating blood and have the potential to revolutionize cancer prevention.[112] To remain relevant, LC NHMs must evolve to integrate biomarker expression levels and demonstrate their effects on disease burden.

Incorporating the natural history of LC recurrence is another key area of opportunity, especially as modern screening and treatment modalities have shifted diagnoses to earlier stages and prolonged survival.[113,114] There is a growing body of literature on modeling and classifying recurrence risk using machine learning; however, these efforts often lack a focus on progression dynamics.[115,116] Expanding LC NHMs by integrating dynamic recurrence models that account for temporal changes in growth requires robust data that captures the specific behaviors of recurrent tumors. This evolution in modeling is crucial for evaluation of effective strategies for treatment and management of LC, ultimately enabling better informed clinical management recommendations [117].

Our study also highlighted the geographic disparity in LC NHM efforts, particularly the underrepresentation of models from low- and middle-income countries.[118] For example, no LC NHM exists for India, a LMIC with a high burden of LC.[119] We acknowledge that the advancement of NHMs is closely tied to the quality of available data, emphasizing the need to enhance cancer registration and standardize clinical trial data globally. Implementing global standards for data collection, particularly in clinical trials, would ensure consistency and comparability across studies. Establishing a Global LC Surveillance Network would significantly advance these efforts by integrating real-time data from cancer registries, clinical trials, and cohort studies, allowing for continuous monitoring of LC from carcinogenesis through recurrence to death.

Despite the increasing availability of novel clinical trial and longitudinal cohort data since the 2000s, only eight (36%) of the 22 models conducted calibration, validation, and sensitivity analysis. The number of models undergoing these critical steps decreased over time. High-quality, longitudinal data are essential for advancing NHMs beyond their current capabilities, yet progress is often hindered by limited data accessibility and outdated infrastructure. Our proposed framework emphasizes the need for robust data-sharing systems that facilitate seamless collaboration between modelers, healthcare providers, and decision-makers. Given that LC NHMs rely heavily on government data to inform key parameters such as cancer incidence and survival rates, modernizing data collection platforms—ideally through co-development by clinicians and modelers—could streamline forecasting pipelines and improve predictive accuracy. Similarly, updating government systems for cancer registry variable recoding and data access would enable more rigorous model evaluation. Expanding computational resources and refining model calibration techniques in parallel would further strengthen the field, ultimately improving the reliability and applicability of LC NHMs.

Standardized reporting and best practices in development of NHMs can strengthen the implementation and increase trust in the evidence-based results generated by LC NHMs. Development of reporting guidelines for NHMs, similar to the CHEERS[120] guidelines for health economics articles, could bolster the rigor and reliability of these models within the research community, thus improving their acceptability of informing clinical and policy decisions.

### Limitations

Our process of collating and summarizing all existing LC NHMs has limitations. Our search was limited to articles published in English, potentially skewing geographic distribution to high-income countries. The current work is also limited to models that met the definition of a NHM model adopted in our study. Models excluded from our review for not meeting the NHM definition used, may also be helpful for comparing cancer modeling approaches. A combination of limitations explicitly written by the authors of each study and implicitly understood from reading model development methodologies were used to inform the expanded LC NHM framework. Therefore, there could be other potential future opportunities LC NHM development that was not included in this review. Finally, there are recommendations in the expanded NHM framework that at the present time are not feasible due to a lack of data, making them more theoretical in nature. For these reasons, it is important to view our findings as general trends in the field and not a definitive perspective on LC NHMs.

## 5. Conclusion

Existing LC NHMs utilized a variety of mathematical techniques and data sources to simulate LC onset and natural disease progression. NHMs must be regularly updated to incorporate new knowledge and to reflect the current LC landscape. Access to data and future research on LC NHMs is needed to enhance modeling accuracy and address existing limitations.

## Supplementary Material

Supplemental Material

## Figures and Tables

**Table 1:** Summary of LC NHMs.

Topic	Group	Count, n (%)	References
NHM Overview (N = 69)	Unique models	22 (32)	[1,25–27,31,34,38,43,45,47,48,52,54,60,63,65,71,74,80,81,87,88]
	Applications/Updates	47 (68)	[12,13,15,16,26,28–30,32,33,35,36,39–42,44,46,49–51,53,55–59,61,62,64,66–70,72,73,75–79,82–86]
	Calibration method description	2 (9)	[33,79]
Methodology (N = 22)	Individual-level	15 (68)	[31,34,35,38,42,43,45,47,54,60,65,74,80,81,88]
	Markov cohort	4 (18)	[48,63,71,87]
	Tumor growth	2 (9)	[1,25]
	Decision tree	1 (5)	[52]
Risk factors (N = 22)	Age	17 (77)	[25,31,34,37,38,43,45,47,48,54,60,63,65,71,74,80,88]
	Smoking history	17 (77)	[1,35,43,45,47,54,56,60–63,65,67,71,80,87,88]
	Sex	17 (77)	[25,35,43,45,47,54,56,60–63,67,71,74,80,87,88]
	Chronic Obstructive Pulmonary Disease	2 (9)	[49,61]
	Human immunodeficiency virus	1 (5)	[64]
	Family and personal history of cancer	1 (5)	[61]
	Body mass index	1 (5)	[61]
	None	2 (9)	[52,81]
	Explicit modeling using a multistage carcinogenesis model	10 (45)	[35,43,45,47,54,56,60,61,67,88]
Smoking history-based risk (N = 22)	Smoking status, intensity, duration, years since cessation (All)	9 (41)	[43,47,54,56,60–62,67,88]
	Smoking status, duration, and years since cessation	1 (5)	[45]
	Smoking status and duration	1 (5)	[63]
	Smoking status and years since cessation	1 (5)	[1]
	Smoking status only	4 (18)	[65,71,80,87]
	Not Reported	1 (5)	[35]
	Never-smoking	6 (27)	[32,45,50,54,60,88]
LC progression (N = 22)	Tumor growth model	10 (45)	[1,25,31,34,38,43,45,54,60,74]
	State transition	12 (55)	[35,42,47,48,52,63,65,71,80,81,87,88]
	Never smokers	3 (14)	[32,45,50]
Tumor growth model (N = 22)	Exponential growth	6 (27)	[1,25,43,45,56,85]
	Gompertzian growth	3 (14)	[31,54,60]
	Exponential and Gompertzian growth	1 (5)	[47]
Tumor growth rate distributions (N = 22)	Log-normal	6 (27)	[1,25,54,60,74]
	Gamma	1 (5)	[45]
	Not Reported	3 (14)	[43,47,62]
Histology subtypes (N = 22)	NSCLC (Any)	14 (64)	[1,35,43,47,48,56,60–62,67,71,81,87,88]
	NSCLC (No subdivision)	4 (18)	[43,48,81,87]
	NSCLC (Subdivision)	10 (45)	[1,35,47,56,60–62,67,71,88]
	SCLC	9 (41)	[1,47,56,60–62,67,71,88]
NSLC subtypes (N = 22)	Adenocarcinoma	5 (23)	[1,47,56,62,71]
	Adenocarcinoma in situ	3 (14)	[47,56,62]
	Large cell carcinoma	5 (23)	[47,56,60–62]
	Squamous cell carcinoma	9 (41)	[1,47,56,60–62,67,71,88]
	Adenocarcinoma and Adenocarcinoma in situ	2 (9)	[60,61]
	Adenocarcinoma and Large cell	2 (9)	[35,88]
	Adenocarcinoma, Large cell, Adenocarcinoma in situ	1 (5)	[67]
	Other NSCLC (Other)	5 (23)	[47,61,62,67,71]
LC staging (NSCLC) (N = 22)	AJCC	7 (32)	[61,63,65,67,71,87,88]
	TNM	3 (14)	[47,62,74]
	SEER	4 (18)	[48,52,54,80]
	Early/Advanced	7 (32)	[1,25,35,43,56,60,81]
	TNM mapped to SEER	1 (5)	[45]
Survival (N = 22)	LC-specific mortality	20 (91)	[1,25,35,43,45,47,52,54,56,60–62,65,67,71,74,80,81,87,88]
	LC stage-specific mortality	11 (50)	[47,52,61,62,65,67,71,74,80,81,88]
	Age stratification	5 (23)	[25,54,61,62,88]
	Sex stratification	7 (32)	[43,54,56,61,62,67,71]
	Competing causes of death	20 (91)	[25,27,31,34,37,38,43,45,47,48,54,60,63,65,71,74,80,81,87,88]

Abbreviations: LC = lung cancer, NHM = natural history model, NSCLC = non-small cell lung cancer, SCLC = small cell lung cancer, AJCC = American Joint Committee on Cancer (ex. I, II, III, IV), TNM = tumor, node, and metastasis (ex. T1N1M1), SEER = Surveillance, Epidemiology, and End Results Program (ex. localized, regional, distant).

**Table 2:** LC NHM common data sources used to inform model parameters.

Data Source	Incidence	Mortality/Survival	Cancer Progression	LC histology and stage
Cancer registry, n (%)	8 (36) [47,54,56,62,63,67,80,88]	8 (36) [31,37,39,47,65,71,88,121]	6 (27) [31,39,45,47,48,55,63]	5 (23) [37,39,45,47,88]
Literature, n (%)	6 (27) [25,58,60,71,80,81]	6 (27) [1,25,52,63,80,88]	2 (9) [1,25]	2 (9) [80,88]
Global data source (Globocan, WHO mortality index), n (%)	2 (9) [80,87]	3 (14) [62,80,81]	NA	1 (5) [81]
NCI Shared resource, n (%)	NA	7 (32) [35,39,45,53,55,78,88]	NA	NA
Carcinogenesis model, n (%)	10 (45) [35,38,43,45,54,56,60,61,67,88]	NA	NA	NA
National statistical agencies/life tables, n (%)	1 (5) [87]	9 (41) [25,48,54,55,60,74,75,80,81]	NA	NA
Mayo Lung Project, n (%)	3 (14) [29,34,43]	3 (14) [29,34,43]	3 (14) [29,34,43]	1 (5) [35]
ELCAP, n (%)	NA	NA	3 (14) [55,75,85]	NA
PLCO, n (%)	5 (22) [47]	5 (22) [47]	5 (22) [47]	5 (22) [47,60]
NLST, n (%)	7 (32) [47,60,75]	5 (22)[47]	7 (32) [47,55,75]	7 (32) [47,60,71]

Abbreviations: LC = lung cancer, Globocan = Global Cancer Observatory, WHO = World Health Organization, NCI = U.S. National Cancer Institute; ELCAP = Early Lung Cancer Action Program, PLCO = Prostate, Lung, Colorectal, and Ovarian Cancer Screening Trial; NLST = National Lung Cancer Screening Trial, NA = not applicable.

* A model is cited in each cell at most one time.

**Table 3:** Current state of LC NH modeling limitations and recommended future steps.

Model Domain	Description	Limitation	Recommended Future Steps
Incidence	Carcinogenesis risk factors	Inconsistent capture of LC risk factorsSecond-hand smoking, genetic predisposition, sociodemographic factors, air pollution, body mass index, and/or occupational exposure risk was not included. [41,48,54,58,62,63,68,71,76,88]Non-smoking related risk factors, non-tobacco smoking. genomic risk profiles or polygenetic risk scores.	1. Establish the relationship between additional risk factors and lung cancer incidence to generate evidence that can inform model parameters. 2. Routinely update risk models to include additional risk factors as new data and evidence becomes available. 3. Develop life tables stratified by non-smoking-related risk factors.
Natural History	Modeling tumor growth	Modeling of tumor growth does not fully reflect complexity of clinical outcomes Constant rate of uniform tumor growth, spherical tumor shape.[1,25,34,43,45,54,60,74,77]Future models should account for dynamic rate of tumor growth and irregular nodule shape.[25,45]	1. Leverage novel tumor measurement approaches: Integrate 3D modeling of tumor size/volume to better capture growth dynamics by type and histology. 2. Variation of growth rate: Develop novel tumor growth models that allow for variation in tumor growth rates to better reflect clinical understanding of LC progression.
		Modeling of tumor growth does not account for non-solid nodulesModels were limited to solid nodules.[31,71]	1. Systematic Collection of Nodule Type Data: Future clinical trials should systematically collect data on nodule characteristics to capture progression of ground-glass and part-solid nodules. 2. Adhering to structured imaging and comprehensive reporting: Imaging data should be recorded in a structured manner that provide comprehensive longitudinal information of the abnormality detected.
		Modeling do not include benign, secondary, and/or metastatic tumor growth (Addressed by n = 2 [45,62]).Limited model to primary tumor.[1,34,43,54,60]	1. Structured collection of lung lesion data in radiology reports, clinical trials, and longitudinal studies. 2. Comprehensive longitudinal data of growth dynamics of lung nodules stratified by sex, histology, and pathological findings.
	Cancer progression stratification	Model relies on data that does not accurately capture population LC dynamics.Limited country or state-specific data for modeling the progression of LC. [80,81,87]	1. Robust cancer registries: Strengthen national and subnational cancer surveillance programs to allow for more relevant analyses. 2. Link registries to epidemiological data: Due to the high reliance on SEER data, including robust information on smoking history would be beneficial.
		LC progression is primarily stratified by sex and/or histology and does not account for other biological influences.Model did not incorporate biological influences on LC NHM beyond histology.	1. Comprehensive biomarker data: Expression levels across different stages of lung cancer stratified by histology, sociodemographic factors, smoking history. 2. Imaging data: Longitudinal imaging data correlating with biomarker expression levels to enhance existing methods of modeling cancer progression. 3. Integrated Biological Modeling: Develop models that integrate biological factors into all aspects of NHMs, including growth rates, transition probabilities, and survival outcomes, to better reflect the impact of these variables on cancer progression.
Recurrence Natural History	Modeling the progression of recurrent tumors	Simulated life-course does not include LC recurrence dynamics. Recurrence or relapse post-curative treatment is captured with limited confidence,[1] assumed to be captured by survival rate,[80] or listed explicitly as a limitation.[67]	1. Longitudinal data on recurrence: Detailed, long-term follow-up data capturing the timing and frequency of cancer recurrence post-treatment, including factors that influence recurrence onset and progression. 2. Detailed Treatment Outcome Data: Comprehensive data on the outcomes of various treatments (surgery, chemotherapy, radiotherapy, immunotherapy) and their impact on recurrence rates, including patient demographics and clinical characteristics.
Model applicability	Temporal and geographic effects	Birth cohort-specific data limits generalizability of NHM findings[16,67,73,76,77];	1. Generation of Geographically Specific and Cross-sectional Data: Establish new cohorts or expand existing cancer registries and health databases to capture detailed geographic-specific data over periods of time. 2. Integrate Modern Migration Patterns: Account for shifts in population risk profiles and healthcare access, improving model accuracy for regions experiencing demographic changes.

Abbreviations: LC = lung cancer, NHM = natural history model, 3D = 3-dimensional, SEER = Surveillance, Epidemiology, and End Results Program.
