# Supplementary material for: Natural history models for lung Cancer: A scoping review
Source: Lung Cancer. Author manuscript; Available in PMC 2025 May 15. (PMC12077999; doi:10.1016/j.lungcan.2025.108495)
Supplement: Supplemental Material [file NIHMS2072770-supplement-Supplemental_Material.pdf]

Supplementary Appendix

TABLE 1: PRISMA SCOPING REVIEW CHECKLIST 2
TABLE 2: LITERATURE SEARCH STRATEGY 4
SUPPLEMENTARY APPENDIX A: REFERENCES OF EXCLUDED ARTICLES 8
TABLE 3: CHARACTERISTICS OF 22 LUNG CANCER NHMS (N = 69) 30
TABLE 4. DATA SOURCES USED TO INFORM, CALIBRATE, AND VALIDATE LC NHMS 35
TABLE 5. CALIBRATION, VALIDATION, AND SENSITIVITY ANALYSIS OF LC NHMS 43

Table 1: PRISMA Scoping Review Checklist

Preferred Reporting Items for Systematic reviews and Meta-Analyses extension for Scoping Reviews (PRISMA-ScR) Checklist

SECTION	ITEM	PRISMA-ScR CHECKLIST ITEM	REPORTED ON PAGE #
**TITLE**			
Title	1	Identify the report as a scoping review.	1
**ABSTRACT**			
Structured summary	2	Provide a structured summary that includes (as applicable): background, objectives, eligibility criteria, sources of evidence, charting methods, results, and conclusions that relate to the review questions and objectives.	2
**INTRODUCTION**			
Rationale	3	Describe the rationale for the review in the context of what is already known. Explain why the review questions/objectives lend themselves to a scoping review approach.	3,4
Objectives	4	Provide an explicit statement of the questions and objectives being addressed with reference to their key elements (e.g., population or participants, concepts, and context) or other relevant key elements used to conceptualize the review questions and/or objectives.	4
**METHODS**			
Protocol and registration	4	Indicate whether a review protocol exists; state if and where it can be accessed (e.g., a Web address); and if available, provide registration information, including the registration number.	5
Eligibility criteria	4	Specify characteristics of the sources of evidence used as eligibility criteria (e.g., years considered, language, and publication status), and provide a rationale.	5
Information sources*	4	Describe all information sources in the search (e.g., databases with dates of coverage and contact with authors to identify additional sources), as well as the date the most recent search was executed.	5
Search	4	Present the full electronic search strategy for at least 1 database, including any limits used, such that it could be repeated.	5
Selection of sources of evidence†	5	State the process for selecting sources of evidence (i.e., screening and eligibility) included in the scoping review.	4
Data charting process‡	5	Describe the methods of charting data from the included sources of evidence (e.g., calibrated forms or forms that have been tested by the team before their use, and whether data charting was done independently or in duplicate) and any processes for obtaining and confirming data from investigators.	5
Data items	5	List and define all variables for which data were sought and any assumptions and simplifications made.	5,6

SECTION	ITEM	PRISMA-ScR CHECKLIST ITEM	REPORTED ON PAGE #
Critical appraisal of individual sources of evidence§	NA	If done, provide a rationale for conducting a critical appraisal of included sources of evidence; describe the methods used and how this information was used in any data synthesis (if appropriate).	NA
Synthesis of results	5	Describe the methods of handling and summarizing the data that were charted.	6
**RESULTS**			
Selection of sources of evidence	5	Give numbers of sources of evidence screened, assessed for eligibility, and included in the review, with reasons for exclusions at each stage, ideally using a flow diagram.	6
Characteristics of sources of evidence	Table 1	For each source of evidence, present characteristics for which data were charted and provide the citations.	Table 1, Table 2, Supp Table 3, Supp Table 4
Critical appraisal within sources of evidence	NA	If done, present data on critical appraisal of included sources of evidence (see item 12).	NA
Results of individual sources of evidence	5-8	For each included source of evidence, present the relevant data that were charted that relate to the review questions and objectives.	Table 1, Table 2, Table 3, Supp Table 3, Supp Table 4, Supp Table 5
Synthesis of results	9	Summarize and/or present the charting results as they relate to the review questions and objectives.	5-9
**DISCUSSION**			
Summary of evidence	9	Summarize the main results (including an overview of concepts, themes, and types of evidence available), link to the review questions and objectives, and consider the relevance to key groups.	9-11
Limitations	11	Discuss the limitations of the scoping review process.	11
Conclusions	11-12	Provide a general interpretation of the results with respect to the review questions and objectives, as well as potential implications and/or next steps.	11
**FUNDING**			
Funding	9	Describe sources of funding for the included sources of evidence, as well as sources of funding for the scoping review. Describe the role of the funders of the scoping review.	12

JBI = Joanna Briggs Institute; PRISMA-ScR = Preferred Reporting Items for Systematic reviews and Meta-Analyses extension for Scoping Reviews.
* Where *sources of evidence* (see second footnote) are compiled from, such as bibliographic databases, social media platforms, and Web sites.
† A more inclusive/heterogeneous term used to account for the different types of evidence or data sources (e.g., quantitative and/or qualitative research, expert opinion, and policy documents) that may be eligible in a scoping review as opposed to only studies. This is not to be confused with *information sources* (see first footnote).
‡ The frameworks by Arksey and O'Malley (6) and Levac and colleagues (7) and the JBI guidance (4, 5) refer to the process of data extraction in a scoping review as data charting.
§ The process of systematically examining research evidence to assess its validity, results, and relevance before using it to inform a decision. This term is used for items 12 and 19 instead of "risk of bias" (which is more applicable to systematic reviews of interventions) to include and acknowledge the various sources of evidence that may be used in a scoping review (e.g., quantitative and/or qualitative research, expert opinion, and policy document).

From: Tricco AC, Lillie E, Zarin W, O'Brien KK, Colquhoun H, Levac D, et al. PRISMA Extension for Scoping Reviews (PRISMAScR): Checklist and Explanation. Ann Intern Med. 2018;169:467–473.

Table 2: Literature Search Strategy

The search strategy was developed by YG, a librarian at the MD Anderson Cancer Center, and RSN.

Database	Search Strings
Medline (OVID)	1 exp Lung Neoplasms/<br>2 ((lung or pulmonary) adj2 (cancer or neoplasm* or carcinoma* or adenocarcinoma*)).ti,ab,kf.<br>3 1 or 2 [Lung Cancer]<br>4 limit 3 to english language<br>5 (animals not (humans and animals)).sh.<br>6 4 not 5<br>7 (animal or mice or mouse or murine or rat or rats or rodent or "in vitro" or "cell line").ti.<br>8 6 not 7 [Remove animal study]<br>9 ("Natural history" or "natural course").ti,ab,kf.<br>10 exp Disease Progression/<br>11 ((tumor or cancer) adj3 (evolution or progression) adj3 model*).ti,ab,kf.<br>12 ("lung cancer development" and model).ab.<br>13 ((disease or clinical) adj2 (trajectory or course* or progression)).ti,ab,kf.<br>14 "Early Detection of Cancer"/ and "lung cancer".ti,kf.<br>15 lung cancer screening.ti.<br>16 or/9-15 [Natural History]<br>17 models, statistical/ or exp models, economic/<br>18 ((Mathematical or Computational or Stochastic or Deterministic or Agent-based or Transition or Markov) adj2 model*).ti,ab,kf.<br>19 (microsimulation or simulation).ti,kf.<br>20 ((microsimulation or simulation) adj2 model*).ab.<br>21 model*.ti,kf.<br>22 Calibration/<br>23 or/17-22 [Modeling]<br>24 8 and 16 and 23<br>25 ((carcinogenesis or progression) adj3 model*).ti,ab,kf.

	26 8 and 25 [lung cancer progression model]<br>27 24 or 26<br>28 exp *Lung Neoplasms/<br>29 ((lung or pulmonary) adj2 (cancer or neoplasm* or carcinoma* or adenocarcinoma*)).ti,kf. or (lung cancer.ab. and ("cancer screening" or nsclc or model* or validation).ti.)<br>30 28 or 29<br>31 8 and 9 and 30 [natural history lung cancer]<br>32 27 or 31 [Final results]<br>33 (editorial or preprint).pt.<br>34 32 not 33
EMBASE (Ovid)	1 exp lung cancer/<br>2 ((lung or pulmonary) adj2 (cancer or neoplasm* or carcinoma* or adenocarcinoma*)).ti,ab,kf.<br>3 1 or 2 [Lung Cancer]<br>4 limit 3 to english language<br>5 Human/<br>6 Nonhuman/ or ANIMAL/ or Animal Experiment/<br>7 6 not 5<br>8 4 not 7<br>9 (animal or mice or mouse or murine or rat or rats or rodent or "in vitro" or "cell line").ti.<br>10 8 not 9 [Remove animal study]<br>11 ("Natural history" or "natural course").ti,ab,kf.<br>12 disease course/ or disease exacerbation/<br>13 ((tumor or cancer) adj3 evolution adj3 model*).ti,ab,kf.<br>14 ("lung cancer development" and model).ab.<br>15 ((disease or clinical) adj2 (trajectory or course* or progression)).ti,ab,kf.<br>16 ((carcinogenesis or progression) adj3 model*).ti,ab,kf.<br>17 cancer screening/ and "lung cancer".ti,kf.<br>18 lung cancer screening.ti.<br>19 or/11-18 [Natural History/Disease progression/cancer screening]<br>20 10 and 19<br>21 statistical model/ or econometric model/

	22 ((Mathematical or Computational or Stochastic or Deterministic or Agent-based or Transition or Markov) adj2 model*).ti,ab,kf.<br>23 ((carcinogenesis or progression) adj3 model*).ti,ab,kf.<br>24 (microsimulation or simulation).ti,kf.<br>25 ((microsimulation or simulation) adj2 model*).ab.<br>26 model*.ti,kf.<br>27 calibration/<br>28 or/21-27 [Modeling]<br>29 20 and 28<br>30 exp *lung cancer/<br>31 ((lung or pulmonary) adj2 (cancer or neoplasm* or carcinoma* or adenocarcinoma*)).ti. or (lung cancer.ab. and ("cancer screening" or nsclc or model* or validation).ti.)<br>32 30 or 31<br>33 29 or (10 and 11 and 32)<br>34 (conference abstract or preprint or editorial).pt.<br>35 33 not 34 [Final results]
Web of Science (Clarivate)	1 TS=(((lung or pulmonary) NEAR/2 (cancer or neoplasm* or carcinoma* or adenocarcinoma*)))<br>2 TS=(((carcinogenesis or progression) NEAR/3 model*))<br>3 #2 AND #1<br>4 TS=("Natural history" OR "Natural course")<br>5 (TI=(("early detection" OR screening))) AND TI=(lung cancer)<br>6 TS=((disease or clinical) NEAR/2 (trajectory or course* or progression))<br>7 #6 OR #5 OR #4<br>8 #7 AND #1<br>9 (TI=(model*)) OR AK=(model*)<br>10 TS=((Mathematical or Computational or Stochastic or Deterministic or Agent-based or Transition or Markov) NEAR/2 model*)<br>11 TS=(microsimulation or simulation)<br>12 #11 OR #10 OR #9<br>13 #12 AND #8<br>14 (TI=("lung cancer")) OR AK=("lung cancer")<br>15 #1 AND #4 AND #14<br>16 #15 OR #13 OR #3

	17 TI=(animal or mice or mouse or murine or rat or rats or rodent or "in vitro" or "cell line")<br>18 #16 NOT #17<br>19 #16 NOT #17 and Meeting Abstract (Exclude – Document Types)<br>20 #16 NOT #17 and Meeting Abstract (Exclude – Document Types) and English (Languages)
IEEE Xplore (NHM)	"41 results for (""Full Text & Metadata"":""natural history"") AND (""Full Text & Metadata"":""lung cancer"") AND (""Full Text & Metadata"":model*) 246 results for (""Full Text & Metadata"":""disease progression"") AND (""Full Text & Metadata"":""lung cancer"") AND (""Full Text & Metadata"":model*) 144 (""Full Text & Metadata"":carcinogenesis OR ""Full Text & Metadata"":tumorgenesis) AND (""Full Text & Metadata"":""lung cancer"") AND (""Full Text & Metadata"":model*) exported results merged into one spreadsheet of 401 records"

Supplementary Appendix A: References of excluded articles

Title	Authors	Year	Journal	Exclusion Reason
Economic impact of lung cancer screening in France: A modeling study	Gendarme, S.; Perrot, E.; Reskot, F.; Bhoowabul, V.; Fourre, G.; Souquet, P. J.; Milleron, B.; Couraud, S.	2017	Revue des Maladies Respiratoires	Article Type
Is our natural-history model of lung cancer wrong?	Bach, P. B.	2008	Lancet Oncology	Article Type
Quantifying and monitoring overdiagnosis in cancer screening: A systematic review of methods	Carter, J. L.; Coletti, R. J.; Harris, R. P.	2015	BMJ (Online)	Article Type
CT screening for lung cancer: Is the evidence strong enough?	Field, J. K.; Devaraj, A.; Duffy, S. W.; Baldwin, D. R.	2016	Lung Cancer	Article Type
BC Cancer Lung Screening Program: Insights on a risk model-based approach for primary care providers	Lam, S.; Sam, J.; Lui, J.; Zhang, Y.; Mayo, J.	2023	British Columbia Medical Journal	Article Type
Epidemiology, prevention, prognostic factors, and natural history of lung cancer	Miller, A. B.	1992	Current Opinion in Oncology	Article Type
On the relationship between tumour growth rate and survival in non-small cell lung cancer	Mistry, H. B.	2017	PeerJ	Article Type
Calibration Methods Used in Cancer Simulation Models and Suggested Reporting Guidelines	Stout, N. K.; Knudsen, A. B.; Kong, C. Y.; McMahon, P. M.; Gazelle, G. S.	2009	Pharmacoeconomics	Article Type
Risk-Based lung cancer screening: A systematic review	Toumazis, I.; Bastani, M.; Han, S. S.; Plevritis, S. K.	2020	Lung Cancer	Article Type
Survival of patients with non-small cell lung cancer without treatment: a systematic review and meta-analysis	Wao, H.; Mhaskar, R.; Kumar, A.; Miladinovic, B.; Djulbegovic, B.	2013	Systematic Reviews	Article Type
Supplementary data for a model-based health economic evaluation on lung cancer screening with low-dose computed tomography in a high-risk population	Du, Y.; Sidorenkov, G.; Heuvelmans, M. A.; Groen, H. J. M.; Vermeulen, K. M.; Greuter, M. J. W.; de Bock, G. H.	2020	Data in Brief	Article Type

Optimizing tobacco advertising bans in seven latin american countries: Microsimulation modeling of health and financial impact to inform evidence-based policy	Bardach, A.; Alcaraz, A.; Roberti, J.; Ciapponi, A.; Augustovski, F.; Pichon-Riviere, A.	2021	International Journal of Environmental Research and Public Health	care delivery
Modelling Health State Utilities as a Transformation of Time to Death in Patients with Non-Small Cell Lung Cancer	Hatswell, A. J.; Chaudhary, M. A.; Monnickendam, G.; Moreno-Koehler, A.; Frampton, K.; Shaw, J. W.; Penrod, J. R.; Lawrance, R.	2023	Pharmacoeconomics	care delivery
A protocol for a cluster randomized trial of care delivery models to improve the quality of smoking cessation and shared decision making for lung cancer screening	Lowenstein, L. M.; Shih, Y. T.; Minnix, J.; Lopez-Olivo, M. A.; Maki, K. G.; Kypriotakis, G.; Leal, V. B.; Shete, S. S.; Fox, J.; Nishi, S. P.; Cinciripini, P. M.; Volk, R. J.	2023	Contemporary Clinical Trials	care delivery
On the optimal policies of cancer screening	Tsodikov, A. D.; Yakovlev, AYu	1991	Mathematical Biosciences	care delivery
Cost-effectiveness of screening smokers and ex-smokers for lung cancer in the Netherlands in different age groups	Al Khayat, M..J.F.H. Eijsink.M.J. Postma.E.M.W. van de Garde. and M. van Hulst	2022	European Journal of Health Economics	detail
Mean sojourn time and effectiveness of mortality reduction for lung cancer screening with computed tomography	Chien, C.R. and T.H. Chen	2008	International Journal of Cancer	detail
Implementing low-dose computed tomography screening for lung cancer in Canada: Implications of alternative at-risk populations, screening frequency, and duration	Evans, W.K..W.M. Flanagan.A.B. Miller.J.R. Goffin.S. Memon.N. Fitzgerald. and M.C. Wolfson	2016	Current Oncology	detail
Clinical impact and cost-effectiveness of integrating smoking cessation into lung	Evans, W.K..C.L. Gauvreau.W.M. Flanagan.S. Memon.J.H.E.	2020	CMAJ open	detail

cancer screening: a microsimulation model	Yong.J.R. Goffin.N.R. Fitzgerald.M. Wolfson. and A.B. Miller			
Estimating the cost of lung cancer diagnosis and treatment in Canada: the POHEM model	Evans, W.K..B.P. Will.J.M. Berthelot. and M.C. Wolfson	1995	Canadian Journal of Oncology	detail
Performance of the cancer risk management model lung cancer screening module	Flanagan, W.M..W.K. Evans.N.R. Fitzgerald.J.R. Goffin.A.B. Miller. and M.C. Wolfson	2015	Health Reports	detail
Cost-Effectiveness of an Organized Lung Cancer Screening Program for Asbestos-Exposed Subjects	Gendarme, S..J.C. Pairon.P. Andujar.F. Laurent.P. Brochard.F. Delva.B. Clin.A. Gislard.C. Paris.I. Thaon.H. Goussault.F. Canoui-Poitrine. and C. Chouaid	2022	Cancers	detail
Modeling the impact of novel systemic treatments on lung cancer screening benefits	Gogebakan, K.C..J. Lange.C.G. Slatore. and R. Etzioni	2023	Cancer	detail
Updated cost-effectiveness analysis of lung cancer screening for Australia, capturing differences in the health economic impact of NELSON and NLST outcomes	Harpaz, S.B..M.F. Weber.S. Wade.P.J. Ngo.P. Vaneckova.P.E.A. Sarich.S. Cressman.M.C. Tammemagi.K. Fong.H. Marshall.A. McWilliams.J.R. Zalcberg.M. Caruana. and K. Canfell	2023	British Journal of Cancer	detail
Cost Utility Analysis of a Pilot Study for the Korean Lung Cancer Screening Project	Kim, J..B. Cho.S.H. Kim.C.M. Choi.Y. Kim. and M.W. Jo	2022	Cancer Research & Treatment	detail
Cost-effectiveness and health impact of lung cancer screening with low-dose computed	Kowada, A.	2022	BMC Pulmonary Medicine	detail

tomography for never smokers in Japan and the United States: a modelling study				
Economic decision analysis model of screening for lung cancer	Marshall, D..K.N. Simpson.C.C. Earle. and C.W. Chu	2001	European Journal of Cancer	detail
Impact of low-dose CT screening for lung cancer on ethnic health inequities in New Zealand: a cost-effectiveness analysis	McLeod, M..P. Sandiford.G. Kvizhinadze.K. Bartholomew. and S. Crengle	2020	BMJ Open	detail
Projected Clinical, Resource Use, and Fiscal Impacts of Implementing Low-Dose Computed Tomography Lung Cancer Screening in Medicare	Roth, J.A..S.D. Sullivan.B.H. Goulart.A. Ravelo.J.C. Sanderson. and S.D. Ramsey	2015	Journal of oncology practice/American Society of Clinical Oncology	detail
Efficacy of Risk Prediction Models and Thresholds to Select Patients for Lung Cancer Screening	Smith, R.J..T. Vijayaharan.V. Linehan.Z. Sun.J.H. Ein Yong.S. Harris.H.H. Mariathas. and R. Bhatia	2022	Canadian Association of Radiologists Journal	detail
Determining cost-effectiveness of lung cancer screening in urban Chinese populations using a state-transition Markov model	Sun, C..X. Zhang.S. Guo.Y. Liu.L. Zhou.J. Shi.N. Wu.Z. Zhai. and G. Liu	2021	BMJ Open	detail
Cost-effectiveness of lung cancer screening combined with nurse-led smoking cessation intervention: A population-based microsimulation study	Yuan, J..Y. Sun.F. Xu.M. Li.M. Fan.C. Zhang.K. Wang.H. Li.X. Bu.X. Yan.J. Wang.J. Ma.G. Zhang.M. Chen. and H. Ren	2022	International Journal of Nursing Studies	detail
Lung cancer screening with low-dose computed tomography: National expenditures and cost-effectiveness	Zeng, X..Z. Zhou.X. Luo. and Q. Liu	2022	Frontiers in Public Health	detail
Cost-Effectiveness Analysis of Risk Factor-Based Lung Cancer Screening Program by Low-Dose Computer Tomography in Current Smokers in China	Zhang, T..X. Chen.C. Li.X. Wen.T. Lin.J. Huang.J. He.N. Zhong.J. Jiang. and W. Liang	2023	Cancers	detail

Cost-Effectiveness of Lung Cancer Screening Using Low-Dose Computed Tomography Based on Start Age and Interval in China: Modeling Study	Zhao, Z..L. Du.Y. Li.L. Wang.Y. Wang.Y. Yang. and H. Dong	2022	JMIR Public Health and Surveillance	detail
Cost-effectiveness of Low-Dose Computed Tomography With a Plasma-Based Biomarker for Lung Cancer Screening in China	Zhao, Z..Y. Wang.W. Wu.Y. Yang.L. Du. and H. Dong	2022	JAMA Network Open	detail
Multistate Statistical Modeling: A Tool to Build a Lung Cancer Microsimulation Model That Includes Parameter Uncertainty and Patient Heterogeneity	Bongers, M. L.; de Ruysscher, D.; Oberije, C.; Lambin, P.; Uyl-de Groot, C. A.; Coupe, V. M.	2016	Medical Decision Making	detail
Estimation of mean sojourn time for lung cancer by chest X-ray screening with a Bayesian approach	Chien, C. R.; Lai, M. S.; Chen, T. H.	2008	Lung Cancer	detail
An actuarial approach to comparing early stage and late stage lung cancer mortality and survival	Goldberg, S. W.; Mulshine, J. L.; Hagstrom, D.; Pyenson, B. S.	2010	Population Health Management	detail
Cost-effectiveness analysis of a lung cancer screening programme in Spain	Gomez-Carballo, N.; Fernandez-Soberon, S.; Rejas-Gutierrez, J.	2022	European Journal of Cancer Prevention	detail
Cost-Effectiveness of Follow-Up for Subsolid Pulmonary Nodules in High-Risk Patients	Hammer, M. M.; Palazzo, L. L.; Paquette, A.; Eckel, A. L.; Jacobson, F. L.; Barbosa, E. M.; Kong, C. Y.	2020	Journal of Thoracic Oncology	detail
Chapter 12: Yale lung cancer model	Holford, T. R.; Ebisu, K.; McKay, L.; Oh, C.; Zheng, T.	2012	Risk Analysis	detail
Stepwise Disease Progression Model of Subsolid Lung Adenocarcinoma with Cystic Airspaces	Jung, W.; Cho, S.; Yum, S.; Chung, J. H.; Lee, K. W.; Kim, K.; Lee, C. T.; Jheon, S.	2020	Annals of Surgical Oncology	detail
Targeted Incentive Programs For Lung Cancer Screening Can Improve Population Health And Economic Efficiency	Kim, D. D.; Cohen, J. T.; Wong, J. B.; Mohit, B.; Fendrick, A. M.; Kent, D. M.; Neumann, P. J.	2019	Health Affairs	detail

**Natural History of Localized and Locally Advanced Atypical Lung Carcinoids after Complete Resection: A Joined French-Italian Retrospective Multicenter Study**	Marciello, F.; Mercier, O.; Ferolla, P.; Scoazec, J. Y.; Filosso, P. L.; Chapelier, A.; Guggino, G.; Monaco, R.; Grimaldi, F.; Pizzolitto, S.; Guigay, J.; de Latour, B. R.; Giuffrida, D.; Longchampt, E.; de Montpreville, V. T.; Fadel, E.; Colao, A.; Planchard, D.; Papotti, M.; Faggiano, A.; Baudin, E.	2018	Neuroendocrinology	detail
Rapid disease progression with delay in treatment of non-small-cell lung cancer	Mohammed, N.; Kestin, L. L.; Grills, I. S.; Battu, M.; Fitch, D. L.; Wong, C. Y. O.; Margolis, J. H.; Chmielewski, G. W.; Welsh, R. J.	2011	International Journal of Radiation Oncology Biology Physics	detail
Clinical and economic impact of current ALK rearrangement testing in Spain compared with a hypothetical no-testing scenario	Nadal, E.; Bautista, D.; Cabez√≥n-Guti√©rrez, L.; Ortega, A. L.; Torres, H.; Carcedo, D.; de Alda, L. R.; Garcia, J. F.; Vieitez, P.; Rojo, F.	2021	BMC Cancer	detail
A stochastic Markov chain model to describe lung cancer growth and metastasis	Newton, P. K.; Mason, J.; Bethel, K.; Bazhenova, L. A.; Nieva, J.; Kuhn, P.	2012	PLoS ONE [Electronic Resource]	detail
Cost-effectiveness of the introduction of low-dose CT screening in Japanese smokers aged 55 to 74 years old	Tabata, H.; Akita, T.; Matsuura, A.; Kaishima, T.; Matsuoka, T.; Ohisa, M.; Awai, K.; Tanaka, J.	2014	Hiroshima Journal of Medical Sciences	detail

Sojourn time and lead time projection in lung cancer screening	Wu, D. F.; Erwin, D.; Rosner, G. L.	2011	Lung Cancer	detail
Quantitative models for lung cancer induced by cigarette smoke	Altshuler, B.	1989	Environmental Health Perspectives	incidence/risk
Radon-induced lung cancer in French and Czech miner cohorts described with a two-mutation cancer model	Brugmans, M. J.; Rispens, S. M.; Bijwaard, H.; Laurier, D.; Rogel, A.; Tomasek, L.; Tirmarche, M.	2004	Radiation & Environmental Biophysics	incidence/risk
A population pharmacodynamic model for lactate dehydrogenase and neuron specific enolase to predict tumor progression in small cell lung cancer patients	Buil-Bruna, N.; Lopez-Picazo, J. M.; Moreno-Jimenez, M.; Martin-Algarra, S.; Ribba, B.; Troconiz, I. F.	2014	AAPS Journal	incidence/risk
Early Prediction of Disease Progression in Small Cell Lung Cancer: Toward Model-Based Personalized Medicine in Oncology	Buil-Bruna, N.; Sahota, T.; Lopez-Picazo, J. M.; Moreno-Jimenez, M.; Martin-Algarra, S.; Ribba, B.; Troconiz, I. F.	2015	Cancer Research	incidence/risk
Dynamics of the risk of smoking-induced lung cancer: a compartmental hidden Markov model for longitudinal analysis	Chadeau-Hyam, M.; Tubert-Bitter, P.; Guihenneuc-Jouyaux, C.; Campanella, G.; Richardson, S.; Vermeulen, R.; De Iorio, M.; Galea, S.; Vineis, P.	2014	Epidemiology	incidence/risk
Using Nonlinear Stochastic Evolutionary Game Strategy to Model an Evolutionary Biological Network of Organ Carcinogenesis Under a Natural Selection Scheme	Chen, B. S.; Tsai, K. W.; Li, C. W.	2015	Evolutionary Bioinformatics Online	incidence/risk
Predicting Lung Cancer Occurrence in Never-Smoking Females in Asia: TNSF-SQ, a Prediction Model	Chien, L. H.; Chen, C. H.; Chen, T. Y.; Chang, G. C.; Tsai, Y. H.; Hsiao, C. F.; Chen, K. Y.; Su, W. C.; Wang, W. C.; Huang, M. S.; Chen, Y. M.; Chen, C. Y.; Liang, S. K.;	2020	Cancer Epidemiology, Biomarkers & Prevention	incidence/risk

	Chen, C. Y.; Wang, C. L.; Lee, M. H.; Chung, R. H.; Tsai, F. Y.; Hu, J. W.; Katki, H. A.; Chatterjee, N.; Chanock, S. J.; Rothman, N.; Lan, Q.; Yang, P. C.; Chen, C. J.; Chang, I. S.; Hsiung, C. A.			
Stochastic state-vector model of radiation carcinogenesis applied to radon-induced lung cancer risk	Crawford-Brown, D. J.; Hofmann, W.; Us, E. P. A.; Univ N Carolina, Dept Environm Sci; Engn, Chapel Hill N. C. U. S. A.	2002	7th International Symposium on the Natural Radiation Environment (NRE-VII)	incidence/risk
Using Clinical Risk Models for Lung Nodule Classification	Deppen, S. A.; Grogan, E. L.	2015	Seminars in Thoracic and Cardiovascular Surgery	incidence/risk
Stochastic population dynamic effects for lung cancer progression	Fakir, H.; Tan, W. Y.; Hlatky, L.; Hahnfeldt, P.; Sachs, R. K.	2009	Radiation Research	incidence/risk
Examination of the role of cigarette smoke in lung carcinogenesis using multistage models	Gaffney, M.; Altshuler, B.	1988	Journal of the National Cancer Institute	incidence/risk
Overdiagnosis in lung cancer screening: Estimates from the German Lung Cancer Screening Intervention Trial	Gonzalez Maldonado, S.; Motsch, E.; Trotter, A.; Kauczor, H. U.; Heussel, C. P.; Hermann, S.; Zeissig, S. R.; Delorme, S.; Kaaks, R.	2021	International Journal of Cancer	incidence/risk
Fitting the two-stage model of carcinogenesis to nested case-control data on the Colorado Plateau uranium miners: dependence on data assumptions	Haylock, R. G.; Muirhead, C. R.	2004	Radiation & Environmental Biophysics	incidence/risk
Longitudinal multistage model for lung cancer incidence, mortality, and CT detected indolent and aggressive cancers	Hazelton, W. D.; Goodman, G.; Rom, W. N.; Tockman, M.; Thornquist, M.;	2012	Mathematical Biosciences	incidence/risk

	Moolgavkar, S.; Weissfeld, J. L.; Feng, Z.			
Biological parameters for lung cancer in mathematical models of carcinogenesis	Jacob, P.; Jacob, V.	2003	Radiation Protection Dosimetry	incidence/risk
Novel computational method for predicting polytherapy switching strategies to overcome tumor heterogeneity and evolution	Jonsson, V. D.; Blakely, C. M.; Lin, L.; Asthana, S.; Matni, N.; Olivas, V.; Pazarentzos, E.; Gubens, M. A.; Bastian, B. C.; Taylor, B. S.; Doyle, J. C.; Bivona, T. G.	2017	Scientific Reports	incidence/risk
Role of tumor-associated neutrophils in regulation of tumor growth in lung cancer development: A mathematical model	Kim, Y.; Lee, D.; Lee, J.; Lee, S.; Lawler, S.	2019	PLoS ONE [Electronic Resource]	incidence/risk
Explainable Machine Learning for Lung Cancer Screening Models	Kobylinska, K.; Orlowski, T.; Adamek, M.; Biecek, P.	2022	Applied Sciences-Basel	incidence/risk
Mathematical model predicts response to chemotherapy in advanced non-resectable non-small cell lung cancer patients treated with platinum-based doublet	Kozlowska, E.; Suwinski, R.; Giglok, M.; Swierniak, A.; Kimmel, M.	2020	PLoS Computational Biology	incidence/risk
Mathematical modeling for mutator phenotype and clonal selection advantage in the risk analysis of lung cancer	Li, L. L.; Zhao, T.; He, X. S.; Yang, X. S.; Tian, T. H.; Zhang, X. A.	2022	Theory in Biosciences	incidence/risk
Stochastic modelling of multistage carcinogenesis and progression of human lung cancer	Li, L.; Tian, T.; Zhang, X.	2019	Journal of Theoretical Biology	incidence/risk
Detailed Analysis and Radiomic Prediction of First Progression Sites of First-Line Targeted Therapy for EGFR-Mutant Lung Adenocarcinoma Patients With Systemic Metastasis	Li, X. Y.; Hou, R. P.; Yu, W.; Zhu, X. R.; Li, H. W.; Yang, Y. D.; Qian, D.; Fu, X. L.	2021	Frontiers in Oncology	incidence/risk
Modelling lung tumour risk in radon-exposed uranium miners using generalizations of the	Little, M.; Haylock, R. G.; Muirhead, C. R.	2002	International Journal of Radiation Biology	incidence/risk

two-mutation model of Moolgavkar, Venzon and Knudson				
Modelling of early lung cancer progression: Influence of growth factor production and cooperation between partially transformed cells	Marciniak-Czochra, A.; Kimmel, M.	2007	Mathematical Models & Methods in Applied Sciences	incidence/risk
A DISCRETE EVENT SIMULATION MODEL TO ESTIMATE POPULATION LEVEL HEALTH AND ECONOMIC IMPACTS OF SMOKING CESSATION INTERVENTIONS	Mayorga, M. E.; Reifsnider, O. S.; Wheeler, S. B.; Kohler, R. E.; North Carolina State Univ Raleigh, Ind Syst Engn Raleigh N. C. U. S. A.; Evidera, Wisconsin Ave Ste Bethesda M. D. U. S. A.; Univ N Carolina, Gillings Sch Global Publ Hlth Hlth Policy; Management, Chapel Hill N. C. U. S. A.; Univ N Carolina, Gillings Sch Global Publ Hlth Hlth Policy; Management, Chapel Hill N. C. U. S. A.; University of North Carolina Chapel Hill; University of North, Carolina; University of North Carolina Chapel, Hill	2014	Winter Simulation Conference	incidence/risk
A modeling analysis to compare eligibility strategies for lung cancer screening in Brazil	Miranda-Filho, A.; Charvat, H.; Bray, F.; Migowski, A.; Cheung, L. C.; Vaccarella, S.; Johansson, M.; Carvalho, A. L.; Robbins, H. A.	2021	EClinicalMedicine	incidence/risk
Mutation and cancer: a model for human carcinogenesis	Moolgavkar, S. H.; Knudson, A. G., Jr.	1981	Journal of the National Cancer Institute	incidence/risk

Multistage carcinogenesis and the fraction at risk	Morgenthaler, S.; Herrero, P.; Thilly, W. G.	2004	Journal of Mathematical Biology	incidence/risk
Reconstructing the patient's natural history from electronic health records	Najafabadipour, M.; Zanin, M.; Rodriguez-Gonzalez, A.; Torrente, M.; Nunez Garcia, B.; Cruz Bermudez, J. L.; Provencio, M.; Menasalvas, E.	2020	Artificial Intelligence in Medicine	incidence/risk
Integrating Speculation Detection and Deep Learning to Extract Lung Cancer Diagnosis from Clinical Notes	Pab√≥n, O. S.; Torrente, M.; Provencio, M.; Rodr√≠guez-Gonzalez, A.; Menasalvas, E.	2021	Applied Sciences-Basel	incidence/risk
Predicting Individualized Lung Disease Progression in Treatment-Naive Patients With Lymphangioleiomyomatosis	Palipana, A. K.; Gecili, E.; Song, S.; Johnson, S. R.; Szczesniak, R. D.; Gupta, N.	2023	Chest	incidence/risk
Cancer Histology and Natural History of Patients with Lung Cancer and Venous Thromboembolism	Ruiz-Artacho, P.; Lecumberri, R.; Trujillo-Santos, J.; Font, C.; Lopez-Nunez, J. J.; Peris, M. L.; Diaz Pedroche, C.; Lobo, J. L.; Lopez Jimenez, L.; Lopez Reyes, R.; Jara Palomares, L.; Pedrajas, J. M.; Mahe, I.; Monreal, M.; The Riete, Investigators	2022	Cancers	incidence/risk
Clinical Characteristics and Outcomes of Patients with Lung Cancer and Venous Thromboembolism	Ruiz-Artacho, P.; Trujillo-Santos, J.; Lopez-Jimenez, L.; Font, C.; Diaz-Pedroche, M. D. C.; Sanchez Munoz-Torrero, J. F.; Peris, M. L.; Skride, A.; Maestre, A.; Monreal, M.	2018	TH Open:Companion Journal to Thrombosis and Haemostasis	incidence/risk

An examination of radiation hormesis mechanisms using a multistage carcinogenesis model	Schollnberger, H.; Stewart, R. D.; Mitchel, R. E.; Hofmann, W.	2004	Nonlinearity in Biology Toxicology Medicine	incidence/risk
Multi-source data approach for personalized outcome prediction in lung cancer screening: update from the NELSON trial	Sidorenkov, G.; Stadhouders, R.; Jacobs, C.; Mohamed Hoesein, F. A. A.; Gietema, H. A.; Nackaerts, K.; Saghir, Z.; Heuvelmans, M. A.; Donker, H. C.; Aerts, J. G.; Vermeulen, R.; Uitterlinden, A.; Lenters, V.; van Rooij, J.; Schaefer-Prokop, C.; Groen, H. J. M.; de Jong, P. A.; Cornelissen, R.; Prokop, M.; de Bock, G. H.; Vliegenthart, R.	2023	European Journal of Epidemiology	incidence/risk
Convolutional Neural Network-Based Diagnostic Model for a Solid, Indeterminate Solitary Pulmonary Nodule or Mass on Computed Tomography	Sun, K.; Chen, S.; Zhao, J.; Wang, B.; Yang, Y.; Wang, Y.; Wu, C.; Sun, X.	2021	Frontiers in Oncology	incidence/risk
Use of a multistage model to predict time trends in smoking induced lung cancer	Swartz, J. B.	1992	Journal of Epidemiology & Community Health	incidence/risk
Some stochastic and state space models of human cancers: Some new approaches	Tan, W. Y.; Zhang, L. J.; Chen, C. W.; Int Inst, Informat; System,; Univ Memphis, Dept Math Sci Memphis T. N. U. S. A.; Us Epa, Natl Ctr Environm Assessment Washington D. C. U. S. A.	2006	10th World Multi-Conference on Systemics, Cybernetics and Informatics/12th International Conference on Information Systems Analysis and Synthesis	incidence/risk
A 50-year projection of lung cancer deaths among Japanese males and potential impact evaluation of anti-smoking measures and screening using a computerized simulation model	Yamaguchi, N.; Mizuno, S.; Akiba, S.; Sobue, T.; Watanabe, S.	1992	Japanese Journal of Cancer Research	incidence/risk

Symptom lead time distribution in lung cancer: natural history and prospects for early diagnosis	Ades, A. E.; Biswas, M.; Welton, N. J.; Hamilton, W.	2014	International Journal of Epidemiology	intervention
Validating impact of pretreatment tumor growth rate on outcome of early-stage lung cancer treated with stereotactic body radiation therapy	Atallah, S.; Le, L. W.; Bezjak, A.; MacRae, R.; Hope, A. J.; Pantarotto, J.	2021	Thoracic Cancer	intervention
Modeling of tumor progression in NSCLC and intrinsic resistance to TKI in loss of PTEN expression	Bidkhori, G.; Moeini, A.; Masoudi-Nejad, A.	2012	PLoS ONE [Electronic Resource]	intervention
Joint longitudinal and time-to-event models for multilevel hierarchical data	Brilleman, S. L.; Crowther, M. J.; Moreno-Betancur, M.; Buros Novik, J.; Dunyak, J.; Al-Huniti, N.; Fox, R.; Hammerbacher, J.; Wolfe, R.	2019	Statistical Methods in Medical Research	intervention
Effects of Random Measurement Error on Lung Cancer Screening Decisions: A Retrospective Cohort-Based Microsimulation Study	Caverly, T. J.; Zhang, X.; Hayward, R. A.; Zhu, J.; Waljee, A. K.	2021	Chest	intervention
Lung cancer screening: Simulations of effects of imperfect detection on temporal dynamics	Chapman, B. E.; Yankelevitz, D. F.; Henschke, C. I.; Gur, D.	2005	Radiology	intervention
Cost-effectiveness and Budgetary Consequence Analysis of Durvalumab Consolidation Therapy vs No Consolidation Therapy After Chemoradiotherapy in Stage III Non-Small Cell Lung Cancer in the Context of the US Health Care System	Criss, S. D.; Mooradian, M. J.; Sheehan, D. F.; Zubiri, L.; Lumish, M. A.; Gainor, J. F.; Reynolds, K. L.; Kong, C. Y.	2019	JAMA Oncology	intervention
Translation of research results to simple estimates of the likely effect of a lung cancer screening programme in the United Kingdom	Duffy, S. W.; Field, J. K.; Allgood, P. C.; Seigneurin, A.	2014	British Journal of Cancer	intervention
Forecasting the impact of stereotactic ablative radiotherapy for early-stage lung cancer on the thoracic surgery workforce	Edwards, J. P.; Datta, I.; Hunt, J. D.; Stefan, K.; Ball, C. G.; Dixon, E.; Grondin, S. C.	2016	European Journal of Cardio-thoracic Surgery	intervention

Modeling progression in radiation-induced lung adenocarcinomas	Fakir, H.; Hofmann, W.; Sachs, R. K.	2010	Radiation & Environmental Biophysics	intervention
Prediction of Treatment Response for Combined Chemo- and Radiation Therapy for Non-Small Cell Lung Cancer Patients Using a Bio-Mathematical Model	Geng, C. R.; Paganetti, H.; Grassberger, C.	2017	Scientific Reports	intervention
Cost-Effectiveness of Treatment Thresholds for Subsolid Pulmonary Nodules in CT Lung Cancer Screening	Hammer, M. M.; Eckel, A. L.; Palazzo, L. L.; Kong, C. Y.	2021	Radiology	intervention
First-line pembrolizumab in PD-L1 positive non-small-cell lung cancer: A cost-effectiveness analysis from the UK health care perspective	Hu, X.; Hay, J. W.	2018	Lung Cancer	intervention
Bayesian lead time estimation for the Johns Hopkins Lung Project data	Jang, H.; Kim, S.; Wu, D.	2013	Journal of Epidemiology and Global Health	intervention
Knowledge-based mechanistic modeling accurately predicts disease progression with gefitinib in EGFR-mutant lung adenocarcinoma	L'Hostis, A.; Palgen, J. L.; Perrillat-Mercerot, A.; Peyronnet, E.; Jacob, E.; Bosley, J.; Duruisseaux, M.; Toueg, R.; Lefevre, L.; Kahoul, R.; Ceres, N.; Monteiro, C.	2023	Npj Systems Biology & Applications	intervention
Persistent pulmonary subsolid nodules with solid portions of 5 mm or smaller: Their natural course and predictors of interval growth	Lee, J. H.; Park, C. M.; Lee, S. M.; Kim, H.; McAdams, H. P.; Goo, J. M.	2016	European Radiology	intervention
The long-term course of ground-glass opacities detected on thin-section computed tomography	Lee, S. W.; Leem, C. S.; Kim, T. J.; Lee, K. W.; Chung, J. H.; Jheon, S.; Lee, J. H.; Lee, C. T.	2013	Respiratory Medicine	intervention
First-line atezolizumab plus chemotherapy in treatment of extensive small cell lung cancer: a cost-effectiveness analysis from China	Li, L. Y.; Wang, H.; Chen, X.; Li, W. Q.; Cui, J. W.	2019	Chinese Medical Journal	intervention
Cost-Effectiveness of Lorlatinib as a First-Line Therapy for Untreated Advanced Anaplastic	Li, S.; Li, J.; Peng, L.; Li, Y.; Wan, X.	2021	Frontiers in Oncology	intervention

Lymphoma Kinase-Positive Non-Small Cell Lung Cancer				
First-Line Durvalumab in Addition to Etoposide and Platinum for Extensive-Stage Small Cell Lung Cancer: A U.S.-Based Cost-Effectiveness Analysis	Lin, S.; Luo, S.; Gu, D.; Li, M.; Rao, X.; Wang, C.; Huang, P.; Xu, X.; Weng, X.	2021	Oncologist	intervention
A multistate modeling and simulation framework to learn dose-response of oncology drugs: Application to bintrafusp alfa in non-small cell lung cancer	Liu, H.; Milenkovic-Grisic, A. M.; Krishnan, S. M.; J√∂nsson, S.; Friberg, L. E.; Girard, P.; Venkatakrishnan, K.; Vugmeyster, Y.; Khandelwal, A.; Karlsson, M. O.	2023	Cpt-Pharmacometrics & Systems Pharmacology	intervention
Verification of the correlation between progression-free survival and overall survival considering magnitudes of survival post- progression in the treatment of four types of cancer	Liu, L. Y.; Yu, H.; Bai, J. L.; Zeng, P.; Miao, D. D.; Chen, F.	2015	Asian Pacific Journal of Cancer Prevention: Apjcp	intervention
Annual number of lung cancer deaths potentially avertable by screening in the United States	Ma, J. M.; Ward, E. M.; Smith, R.; Jemal, A.	2013	Cancer	intervention
Cost-benefit analysis of ALK diagnosis vs. non-diagnosis in patients with advanced non-small cell lung cancer in Spain	Majem, M.; Alvarez, R.; Ortega, A. L.; Ruiz de Alda, L.; Gordo, R.; Garcia, J. F.; Ivanova-Markova, Y.; Gonzalez-Dominguez, A.; San Cristobal, R. S.; Rojo, F.	2022	Global & Regional Health Technology Assessment	intervention
Cost-effectiveness analysis of screening for lung cancer with low dose spiral CT (computed tomography) in the Australian setting	Manser, R.; Dalton, A.; Carter, R.; Byrnes, G.; Elwood, M.; Campbell, D. A.	2005	Lung Cancer	intervention
Shall We Screen Lung Cancer With Low-Dose Computed Tomography? Cost-Effectiveness in Hungary	Nagy, B.; Szilberhorn, L.; Gyorbiro, D. M.; Moizs, M.; Bajzik, G.; Kerpel-Fronius, A.; Voko, Z.	2023	Value in Health Regional Issues	intervention

Dose broadening due to target position variability during fractionated breath-held radiation therapy	O'Dell, W. G.; Schell, M. C.; Reynolds, D.; Okunieff, P.	2002	Medical Physics	intervention
Cost-effectiveness of lung cancer screening in Japan	Okamoto, N.	2000	Cancer	intervention
The health and economic burden of smoking in 12 Latin American countries and the potential effect of increasing tobacco taxes: an economic modelling study	Pichon-Riviere, A.; Alcaraz, A.; Palacios, A.; Rodriguez, B.; Reynales-Shigematsu, L. M.; Pinto, M.; Castillo-Riquelme, M.; Pena Torres, E.; Osorio, D. I.; Huayanay, L.; Loza Munarriz, C.; de Miera-Juarez, B. S.; Gallegos-Rivero, V.; De La Puente, C.; del Pilar Navia-Bueno, M.; Caporale, J.; Roberti, J.; Virgilio, S. A.; Augustovski, F.; Bardach, A.	2020	The Lancet Global Health	intervention
Estimating stage-specific sensitivity for cancer screening tests	Pinsky, P.; Lange, J.; Etzioni, R.	2023	Journal of Medical Screening	intervention
Cost-Effectiveness of Lorlatinib for the Treatment of Adult Patients with Anaplastic Lymphoma Kinase Positive Advanced Non-Small Cell Lung Cancer in Spain	Presa, M.; Vicente, D.; Calles, A.; Salinas-Ortega, L.; Naik, J.; Garcia, L. F.; Soto, J.	2023	Clinicoeconomics & Outcomes Research	intervention
Clinical and economic impact of '<i>ROS1-testing'</i> strategy compared to a <i>'no-ROS1-testing'</i> strategy in advanced NSCLC in Spain	Rojo, F.; Conde, E.; Torres, H.; Cabez√≥n-Guti√©rrez, L.; Bautista, D.; Ramos, I.; Carcedo, D.; Arrabal, N.; Garc√≠a, J. F.; Gal√°n, R.; Nadal, E.	2022	BMC Cancer	intervention
Microsimulation modeling of extended annual CT screening	Schreuder, A.; Mets, O. M.; Schaefer-Prokop,	2021	Lung Cancer	intervention

among lung cancer cases in the National Lung Screening Trial	C. M.; Jacobs, C.; Prokop, M.			
Cost-effectiveness of a new autoantibody test added to Computed Tomography (CT) compared to CT surveillance alone in the diagnosis of lung cancer amongst patients with indeterminate pulmonary nodules	Sutton, A. J.; Sagoo, G. S.; Jackson, L.; Fisher, M.; Hamilton-Fairley, G.; Murray, A.; Hill, A.	2020	PLoS ONE [Electronic Resource]	intervention
Pharmacoeconomic analysis of consolidation therapy with pemetrexed after first-line chemotherapy for non-small cell lung cancer	Tsuchiya, T.; Fukuda, T.; Furuiye, M.; Kawabuchi, K.	2011	Lung Cancer	intervention
TUMOR DOUBLING TIME AND PROGNOSTIC ASSESSMENT OF PATIENTS WITH PRIMARY LUNG-CANCER	Usuda, K.; Saito, Y.; Sagawa, M.; Sato, M.; Kanma, K.; Takahashi, S.; Endo, C.; Chen, Y.; Sakurada, A.; Fujimura, S.	1994	Cancer	intervention
Lung cancer from radon: a two-stage model analysis of the WISMUT Cohort, 1955-1998	van Dillen, T.; Dekkers, F.; Bijwaard, H.; Kreuzer, M.; Grosche, B.	2011	Radiation Research	intervention
A cost-utility analysis of lung cancer screening and the additional benefits of incorporating smoking cessation interventions	Villanti, A. C.; Jiang, Y.; Abrams, D. B.; Pyenson, B. S.	2013	PLoS ONE [Electronic Resource]	intervention
Maintenance erlotinib in advanced nonsmall cell lung cancer: cost-effectiveness in EGFR wild-type across Europe	Walleser, S.; Ray, J.; Bischoff, H.; Vergnenegre, A.; Rosery, H.; Chouaid, C.; Heigener, D.; de Castro Carpeno, J.; Tiseo, M.; Walzer, S.	2012	Clinicoeconomics & Outcomes Research	intervention
The natural history of lung cancer estimated from the results of a randomized trial of screening	Walter, S. D.; Kubik, A.; Parkin, D. M.; Reissigova, J.; Adamec, M.; Khlat, M.	1992	Cancer Causes & Control	intervention
Lifespan and medical expenditure prognosis for cancer metastasis - a simulation	Wang, K. J.; Lukito, H.	2023	Computer Methods and Programs in Biomedicine	intervention

modeling using semi-Markov process				
Cost-effectiveness of serplulimab as first-line therapy for extensive-stage small cell lung cancer in China	Xiang, G.; Jiang, T.; Gan, L.; Wu, Y.; Zhang, N.; Xing, H.; Su, H.; Li, Y.; Peng, D.; Ni, R.; Liu, Y.	2023	Frontiers in Immunology	intervention
Evaluation of cancer prevention strategies by computerized simulation model: an approach to lung cancer	Yamaguchi, N.; Tamura, Y.; Sobue, T.; Akiba, S.; Ohtaki, M.; Baba, Y.; Mizuno, S.; Watanabe, S.	1991	Cancer Causes & Control	intervention
Evaluation of cancer prevention strategies by computerized simulation model: methodological issues	Yamaguchi, N.; Tamura, Y.; Sobue, T.; Akiba, S.; Ohtaki, M.; Baba, Y.; Mizuno, S.; Watanabe, S.	1994	Environmental Health Perspectives	intervention
Association of cancer screening and residing in a coal-polluted East Asian region with overall survival of lung cancer patients: a retrospective cohort study	Yang, R. X.; He, M.; Wang, D. M.; Ye, R. R.; Li, L.; Deng, R. Y.; Shah, M.; Yeung, S. C. J.	2020	Scientific Reports	intervention
Long-term follow-up of persistent pulmonary subsolid nodules: Natural course of pure, heterogeneous, and real part-solid ground-glass nodules	Zhang, Z.; Zhou, L.; Min, X.; Li, H.; Qi, Q.; Sun, C.; Sun, K.; Yang, F.; Li, X.	2023	Thoracic Cancer	intervention
Cost-effectiveness analysis of atezolizumab plus chemotherapy in the first-line treatment of extensive-stage small-cell lung cancer	Zhou, K.; Zhou, J.; Huang, J.; Zhang, N.; Bai, L.; Yang, Y.; Li, Q.	2019	Lung Cancer	intervention
Cost-Effectiveness of Artificial Intelligence Support in Computed Tomography-Based Lung Cancer Screening	Ziegelmayer, S.; Graf, M.; Makowski, M.; Gawlitza, J.; Gassert, F.	2022	Cancers	intervention
Unified tumor growth mechanisms from multimodel inference and dataset integration	Beik, S. P.; Harris, L. A.; Kochen, M. A.; Sage, J.; Quaranta, V.; Lopez, C. F.	2023	PLoS Computational Biology	no forecasting
Natural history of pure ground-glass opacity lung nodules detected by low-dose CT scan	Chang, B.; Hwang, J. H.; Choi, Y. H.; Chung, M. P.; Kim, H.; Kwon, O. J.; Lee, H. Y.; Lee, K.	2013	Chest	no forecasting

	S.; Shim, Y. M.; Han, J.; Um, S. W.			
Multistate models for the natural history of cancer progression	Cheung, L. C.; Albert, P. S.; Das, S.; Cook, R. J.	2022	British Journal of Cancer	no forecasting
Turning gray: the natural history of lung cancer over time	Detterbeck, F. C.; Gibson, C. J.	2008	Journal of Thoracic Oncology: Official Publication of the International Association for the Study of Lung Cancer	no forecasting
The growth rate in the interpretation of the natural history of lung cancer	Galante, E.; Reduzzi, D.; Gallus, G.; Guzzon, A.; Cataldo, I.; Valente, M.; Ravasi, G.	1984	Tumori	no forecasting
Model Calibration of Pharmacokinetic-Pharmacodynamic Lung Tumour Dynamics for Anticancer Therapies	Ghita, M.; Billiet, C.; Copot, D.; Verellen, D.; Ionescu, C. M.	2022	Journal of Clinical Medicine	no forecasting
Modelling tumour biology-progression relationships in screening trials	Ghosh, D.	2006	Statistics in Medicine	no forecasting
**The natural history of recurrence after bronchoplastic procedures for non-small cell lung cancer**	Hollaus, P. H.; Wurnig, P. N.; Pridun, N. S.	2003	Annals of Thoracic Surgery	no forecasting
Natural History of Pulmonary Subsolid Nodules: A Prospective Multicenter Study	Kakinuma, R.; Noguchi, M.; Ashizawa, K.; Kuriyama, K.; Maeshima, A. M.; Koizumi, N.; Kondo, T.; Matsuguma, H.; Nitta, N.; Ohmatsu, H.; Okami, J.; Suehisa, H.; Yamaji, T.; Kodama, K.; Mori, K.; Yamada, K.; Matsuno, Y.; Murayama, S.; Murata, K.	2016	Journal of Thoracic Oncology: Official Publication of the International Association for the Study of Lung Cancer	no forecasting
Evaluating the number of stages in development of squamous cell and adenocarcinomas	Kravchenko, J.; Akushevich, I.;	2012	PLoS ONE [Electronic Resource]	no forecasting

across cancer sites using human population-based cancer modeling	Abernethy, A. P.; Lyerly, H. K.			
HER2 insertion YVMA mutant lung cancer: Long natural history and response to afatinib	Li, B. T.; Lee, A.; O'Toole, S.; Cooper, W.; Yu, B.; Chaft, J. E.; Arcila, M. E.; Kris, M. G.; Pavlakis, N.	2015	Lung Cancer	no forecasting
A habituation memristor model for lung cancer screening application	Li, X.; Feng, Z.; Fang, X. Q.; Wu, Z. H.; Zhu, Y. L.; Xu, Z. Y.; Dai, Y. H.	2023	Physica Scripta	no forecasting
Spreaders and sponges define metastasis in lung cancer: a Markov chain Monte Carlo mathematical model	Newton, P. K.; Mason, J.; Bethel, K.; Bazhenova, L.; Nieva, J.; Norton, L.; Kuhn, P.	2013	Cancer Research	no forecasting
Natural history of skeletal-related events in patients with breast, lung, or prostate cancer and metastases to bone: a 15-year study in two large US health systems	Oster, G.; Lamerato, L.; Glass, A. G.; Richert-Boe, K. E.; Lopez, A.; Chung, K.; Richhariya, A.; Dodge, T.; Wolff, G. G.; Balakumaran, A.; Edelsberg, J.	2013	Supportive Care in Cancer	no forecasting
Natural history of pathologically confirmed pulmonary subsolid nodules with deep learning-assisted nodule segmentation	Qi, L. L.; Wang, J. W.; Yang, L.; Huang, Y.; Zhao, S. J.; Tang, W.; Jin, Y. J.; Zhang, Z. W.; Zhou, Z.; Yu, Y. Z.; Wang, Y. Z.; Wu, N.	2021	European Radiology	no forecasting
Lung cancer growth dynamics	Reich, J. M.; Kim, J. S.	2011	European Journal of Radiology	no forecasting
Natural history and clinical characteristics of multiple pulmonary nodules with ground glass opacity	Sato, Y.; Fujimoto, D.; Morimoto, T.; Uehara, K.; Nagata, K.; Sakanoue, I.; Hamakawa, H.; Takahashi, Y.; Imai, Y.; Tomii, K.	2017	Respirology	no forecasting
Variation in experts' beliefs about lung cancer growth, progression, and prognosis	Schultz, E. M.; Silvestri, G. A.; Gould, M. K.	2008	Journal of Thoracic Oncology: Official Publication of the International	no forecasting

			Association for the Study of Lung Cancer	
A Bayesian model for estimating multi-state disease progression	Shen, S.; Han, S. X.; Petousis, P.; Weiss, R. E.; Meng, F.; Bui, A. A.; Hsu, W.	2017	Computers in Biology & Medicine	no forecasting
Differential diagnosis of small cell neuroendocrine carcinoma of the lung	Warren, W. H.; Gould, V. E.	1997	Chest Surgery Clinics of North America	no forecasting
Implications of tumor growth rate for the natural history of lung cancer	Weiss, W.	1984	Journal of Occupational Medicine	no forecasting
Modeling tumor progression via the comparison of stage-specific graphs	Wong, S. W. H.; Pastrello, C.; Kotlyar, M.; Faloutsos, C.; Jurisica, I.	2018	Methods (Duluth)	no forecasting
Prediction of Interval Growth of Lung Adenocarcinomas Manifesting as Persistent Subsolid Nodules <=3 cm Based on Radiomic Features	Wu, F. Z.; Wu, Y. J.; Chen, C. S.; Tang, E. K.	2023	Academic Radiology	no forecasting
Clinical features of 5,628 primary lung cancer patients: experience at Mayo Clinic from 1997 to 2003	Yang, P.; Allen, M. S.; Aubry, M. C.; Wampfler, J. A.; Marks, R. S.; Edell, E. S.; Thibodeau, S.; Adjei, A. A.; Jett, J.; Deschamps, C.	2005	Chest	no forecasting
Description of an incidence-based model for Assessing comorbidity patterns in disease natural history	Kiri, V. A.	2016	BMJ Open	not lung
Mathematical Oncology to Cancer Systems Medicine: Translation from Academic Pursuit to Individualized Therapy with MORA	Majumder, D.	2023	Current Cancer Therapy Reviews	not lung
The economic impact of health policy interventions	Manton, K. G.; Stallard, E.; Tolley, H. D.	1983	Risk Analysis	not lung
Bayesian analysis of a disability model for lung cancer survival	Armero, C.; Cabras, S.; Castellanos, M. E.; Perra, S.; Quiros, A.; Oruezabal, M.	2016	Statistical Methods in Medical Research	survival

	J.; Sanchez-Rubio, J.			
Natural Disease History, Outcomes, and Co-mutations in a Series of Patients With BRAF-Mutated Non-small-cell Lung Cancer	Myall, N. J.; Henry, S.; Wood, D.; Neal, J. W.; Han, S. S.; Padda, S. K.; Wakelee, H. A.	2019	Clinical Lung Cancer	survival
Clinical features and survival of lung cancer patients with pleural effusions	Porcel, J. M.; Gasol, A.; Bielsa, S.; Civit, C.; Light, R. W.; Salud, A.	2015	Respirology	survival
Two-level Bayesian interaction analysis for survival data incorporating pathway information	Qin, X.; Ma, S. G.; Wu, M. Y.	2022	Biometrics	survival
The Natural History of Operable Non-Small Cell Lung Cancer in the National Cancer Database	Rosen, J. E.; Keshava, H. B.; Yao, X.; Kim, A. W.; Detterbeck, F. C.; Boffa, D. J.	2016	Annals of Thoracic Surgery	survival

Table 3: Characteristics of 22 lung cancer NHMs (n = 69)

Model Name* (Original Year)	Modeling Approach	Country	Risk Model (1) and Factors (2)	Progression Factor(s)	Histology	Disease-specific mortality factors	Staging	References
Geddes (1979)[1]	T	UK	Smoking (S, C)	Histology, VDT	NSCLC (AD, SQ) SCLC†, Undifferentiated, Miscellaneous	Tumor size	E/A	[1]
Manton (1982)[25]	T	USA	Age	VDT	NA	Age, Tumor growth latency	E/A	[25]
MISCAN-Lung (1985)[26,37]	M	USA	1) TSCE 2) Age, Sex, Smoking (S, D, I, C), Diet	Stage, Histology	NSCLC (AD/LCC/AIS‡, SQ, O), SCLC	Sex, Histology, Stage	AJCC	[12,13,15,16,26,37,40,46,47,50,51,57,59,66,67,69,72,76,122,123]
Flehinger (1987)[27]	M	USA	1) TSCE 2) Sex, Smoking (NR)	NA	NSCLC (AD/LCC)	Disease latency	E/A	[27-29,35,124]
Lung Cancer Policy Model (2009)[31,33]	M, T	USA	Age, Sex, Smoking (S, D, I, C), Family history of LC, COPD, HIV	Stage, Histology, growth rate decay	NSCLC (AD, LCC, SQ, AIS, O), SCLC	Age, Sex, Stage, Histology, Tumor size, Tumor	TNM	[12,13,15,16,31-33,36,40,41,44,46,47,49,53,57,58,62,64,68,69,76]

Model Name* (Original Year)	Modeling Approach	Country	Risk Model (1) and Factors (2)	Progression Factor(s)	Histology	Disease-specific mortality factors	Staging	References
						metastasis		
Lung Cancer Outcomes Simulator (2009)[34]	M, T	USA	1) TSCE 2) Age, Sex, Smoking (S, D, I, C)	Stage, Histology, Sex, VDT, Tumor metastasis	NSCLC (AD, LCC, SQ, AIS[b]), SCLC	Sex, Tumor size, Tumor metastasis	E/A	[12,13,15,16,34,39,40,46,47,56,57,69,70,76,77,82,83]
Longitudinal Multistage Observation Model (2014)[38,47]	M, T	USA	1) Longitudinal multistage observation by histology 2) Age, Sex, Smoking (S, D, I, C)	Stage, Histology, Tumor growth, Tumor metastasis	NSCLC (AD, LCC, SQ, AIS[b], O), SCLC	Histology, Stage, Tumor size	TNM	[12,38,40,46,47,57]
Goldwasser (2013)[43]	M, T	USA	1) TSCE 2) Age, Sex, Smoking (S, D, I, C)	VDT	NSCLC	Sex, Tumor size	E/A	[43,55]
Chen (2014)[45]	M, T	USA	1) Rice-MD Anderson TSCE model	Tumor growth rate, Metastasized tumor	NA	Primary tumor size, Tumor metastasis	TNM, SEER	[45]

Model Name* (Original Year)	Modeling Approach	Country	Risk Model (1) and Factors (2)	Progression Factor(s)	Histology	Disease-specific mortality factors	Staging	References
			2) Age, Sex, Smoking (S, D, I)	growth rate				
UM-LCSs (2014)[47]	M	USA	1) Multistage clonal expansion model<br>2) Age, Sex, BMI, COPD, Smoking (S, D, I, C), Family and personal history of lung cancer	Histology, Stage at diagnosis	NSCLC (AD/AIS, LCC, SQ, O), SCLC	Age at diagnosis, Sex, Stage, Histology	AJCC	[12,13,15,16,46,47,57,61,69,73,76,78,84,86]
Hinde (2015)[48]	MC	UK	Age	NR	NSCLC	NR	SEER	[48]
Shih (2016)[52]	D	USA	NA	NA	NA	Stage	SEER	[52]
The Microsimulation Lung Cancer model (2017)[54,79]	M, T	USA	1) Smoking-based TSCE<br>2) Age, Sex, Smoking	Tumor growth rate	NA	Age, Sex, Tumor size	SEER	[54,79]

Model Name* (Original Year)	Modeling Approach	Country	Risk Model (1) and Factors (2)	Progression Factor(s)	Histology	Disease-specific mortality factors	Staging	References
			(S, D, I, C)					
Treskova (2017)[60]	M, T	Germany	1) TSCE 2) Age, Sex, Smoking (S, D, I, C)	Histology, Tumor growth rate	NSCLC (AD/AIS, LCC, SQ), SCLC	Tumor volume, Histology	E/A	[60]
Hofer (2018)[63]	MC	Germany	Age, Smoking (S, D)	NR	NA	NR	AJCC	[63]
Exeter NAtural history-Based economic model of Lung cancer screening (2018)[65]	M	UK	Age, Sex, Smoking (S)	NA	NA	Stage	AJCC	[65,75]
Allen (2020)[71]	MC	USA	Age, Sex, Smoking (S)	Histology, Stage, Fast/Slow growing cancer type	NSCLC (AD, SQ, O), SCLC	Sex, Histology, Stage	AJCC	[71]
SiMRiSc Lung (2020)[74]	M, T	Netherlands	Age, Sex	VDT, Tumor metastasis	NA	Stage	TNM	[74,85]
Diaz (2021)[80]	M	Spain	Age, Smoking (S)	NA	NA	Stage	SEER	[80]

Model Name* (Original Year)	Modeling Approach	Country	Risk Model (1) and Factors (2)	Progression Factor(s)	Histology	Disease-specific mortality factors	Staging	References
Esmaeili (2021)[81]	M	Iran	NA	NA	NSCLC	Stage	E/A	[81]
Souliotis (2022)[87]	MC	Greece	Sex, Smoking (S)	Histology, Stage	NSCLC, SCLC	Histology, Stage	AJCC	[87]
NCC-lung (2023)[88]	M	China	1) TSCE 2) Age, Sex, Smoking (S, D, I, C)	Histology	NSCLC (AD/LCC, SQ) SCLC	Age, Stage	AJCC	[88]

Abbreviations: Modeling approach (T = Tumor growth model; MC: Markov Cohort Model; M = Microsimulation; D = Decision Tree; O = Other stochastic process), Smoking (S = Status; D = Duration; I = Intensity; C = Years since cessation from smoking), Histology (NSCLC = Non-Small Cell Lung Carcinoma; SCLC = Small Cell Lung Carcinoma; AD = Adenocarcinoma; SQ = Squamous Cell Carcinoma; LCC = Large Cell Carcinoma; AIS = Adenocarcinoma in situ; LMO = Longitudinal multistage observation by histology), TSCE = two-stage clonal expansion model, Stage (AJCC = American Joint Committee on Cancer staging system; SEER = Surveillance, Epidemiology, and End Results Program staging system; TNM = Tumor, node, metastasis categories; E/A = Early and advanced), NA = not applicable, NR = not reported, VDT = Volume doubling time.

* If no model name is provided, the last name of the primary author was provided.

† Oat Cell carcinoma was written in the original article but was recoded to SCLC.

‡ Bronchioloalveolar Carcinoma was written in the original article but was recoded to Adenocarcinoma in Situ

Table 4. Data sources used to inform, calibrate, and validate LC NHMs

Model Name	Incidence	Prevalence	Mortality (all-cause)	Mortality (DCoD)	Mortality (OCoD	Cancer Progression	Histology Dist.	Stage Dist.	Recur
Geddes (1979)	NA	NA	NA	Lit (Breur 1966; Schwartz 1961; Spratt 1964)<br>***Valid:*** Lit (Doll 1976, Hyde 1976)	NA	VDT: Lit (Brenner 1967; Breur 1966; Chahinian 1972; Garland 1963; Garland 1966; Meyer 1973; Schwartz 1961; Steele 1973; Weidd 1966; Weiss 1974)	Lit (Breur 1966; Schwartz 1961; Spratt 1964)	NA	Lit (Weiss 1966; Meyer 1973)
Manton (1982)	Lit (Manton 1982)	NA	NA	National Center for Health Statistics, Lit (Axtell 1976)	National Center for Health Statistics	***Valid:*** Lit (Steel 1966)	NA	NA	NA
MISCAN-Lung (1985)	2011:<br>***Calib:*** SEER (NR, 1975-1979)<br>2012: TSCE<br>***Calib:*** SEER<br>***Valid:*** PLCO<br>2014:<br>***Calib:*** NLST, PLCO	NA	NA	2011:<br>***Calib:*** SEER (NR)<br>2012: SEER<br>2014:<br>SEER<br>***Calib:*** NLST, PLCO<br>***Valid:*** PLCO	2012: NCI Smoking History Generator	NA	2011:<br>***Calib:*** SEER<br>2014:<br>***Calib:*** SEER, NLST, PLCO<br>***Valid:*** PLCO<br>2014:<br>***Calib:***	2011:<br>***Calib:*** SEER<br>2012:<br>***Valid:*** SEER, PLCO<br>2014:<br>***Calib:*** NLST, PLCO, Ontario Cancer	NA

Model Name	Incidence	Prevalence	Mortality (all-cause)	Mortality (DCoD)	Mortality (OCoD	Cancer Progression	Histology Dist.	Stage Dist.	Recur
	Valid: PLCO<br>2017:<br>***Calib:*** Ontario Cancer Registry<br>2018:<br>***Valid:***<br>Swiss cancer registry			2015:<br>***Valid:*** Lit (Thun 2008)<br>2017:<br>***Calib:***<br>Ontario Cancer Registry<br>2018:<br>***Valid:*** Swiss cancer registry<br>2019:<br>SEER			Ontario Cancer Registry<br>2015:<br>Lit (Subramania n 2007)	Registry<br>***Valid:*** PLCO	
Flehinger 1987	1987:<br>MSKCC LCSP<br>1988:<br>MSKCC LCSP, Johns Hopkins Lung Project<br>1993:<br>MLP<br>2010:<br>TSCE (Meza 2008)<br>***Calib:*** MLP	NA	NA	1987:<br>MSKCC LCSP<br>1988:<br>MSKCC LCSP, Johns Hopkins Lung Project<br>1993:<br>MLP<br>2010:<br>***Calib:*** MLP	1987:<br>MSKCC LCSP<br>1988:<br>MSKCC LCSP, Johns Hopkins Lung Project<br>1993:<br>MLP<br>2010:<br>CISNET Smoking HR<br>***Calib:*** MLP	NA	NA	2010:<br>***Calib:*** MLP	NA
Lung Cancer Policy Model	2008: National health interview survey; NHANES III<br>***Calib:*** SEER<br>***Valid:*** Mayo CT<br>2014:	2015:<br>***Calib:***<br>COPD Prevalence: FOS-LDS	NA	2008:<br>***Calib:*** SEER<br>2014:<br>SEER (17, 1973–2008<br>***Calib:***<br>NLST, PLCO<br>2016:	2009:<br>***Calib:***<br>Lit (Thun 1997; Capwell 1991; Damber 1986; Beadsmoore	2008:<br>Tumor growth: SEER, Lit (McMahon 2006)<br>***Calib:***<br>SEER	***Calib:*** SEER Lit (Kong 2014)<br>2018:<br>***Calib:*** Lit (Sigel 2012; Sigel 2013)	2014<br>***Calib:*** SEER; Lit (Sigel 2012; Sigel 2013)	NA

Model Name	Incidence	Prevalence	Mortality (all-cause)	Mortality (DCoD)	Mortality (OCoD	Cancer Progression	Histology Dist.	Stage Dist.	Recur
	Calib: NLST, PLCO<br>***Valid:*** PLCO<br>2016:<br>***Calib:*** Pennsylvania Cancer Registry<br>2017:<br>***Calib:*** Lit (Kong 2014)<br>2018:<br>***Calib:*** HIV and non-HIV incidence ratio (Sigel 2012; Silverberg 2009; Yanik 2016)			***Calib:*** Pennsylvania Cancer Registry<br>2017:<br>***Calib:*** GLOBOCAN<br>2018:<br>HIV Hazard ratio: Lit (Coghill 2015)<br>2019:<br>GLOBOCAN<br>2019b:<br>***Calib:*** SEER	2003; Beckles 2003)<br>2012:<br>NCI Smoking History Generator<br>2014:<br>***Calib:*** NLST<br>2017:<br>Global Burden of Disease<br>2018:<br>HIV-specific and other mortality from several cohorts, ART-adherence based mortality (Braithwaite 2010; Khademi 2014)<br>2019:<br>Global Burden of Disease				
Lung Cancer Outcomes Simulator (2009)	2009:<br>MLP<br>2014:<br>TSCE (Meza 2008)<br>***Calib:*** SEER,	NA	NA	2009:<br>MLP<br>2012:<br>SEER<br>***Valid:*** MLP<br>2014:	2014:<br>***Calib:*** NLST<br>***Valid:*** PLCO	2009:<br>MLP<br>2012:<br>SEER	2012:<br>SEER<br>2014:<br>***Calib:*** NLST, PLCO	2009:<br>MLP<br>2012:<br>SEER<br>2014:<br>***Calib:*** NLST,	NA

Model Name	Incidence	Prevalence	Mortality (all-cause)	Mortality (DCoD)	Mortality (OCoD	Cancer Progression	Histology Dist.	Stage Dist.	Recur
	NLST, PLCO<br>***Valid:*** PLCO			***Calib:*** NLST, PLCO<br>***Valid:*** PLCO<br>2019:<br>***Calib:*** SEER				PLCO<br>***Valid:*** PLCO	
Longitudinal Multistage Observation Model (FHCRC)	2012:<br>***Calib:*** Carotene and Retinol Efficacy Trial<br>2014:<br>***Calib:*** NLST, PLCO<br>***Valid:*** PLCO	NA	NA	2012:<br>***Calib:*** Carotene and Retinol Efficacy Trial<br>2014:<br>***Calib:*** NLST, PLCO<br>***Valid:*** PLCO	2014:<br>NLST, PLCO	2012<br>***Calib:*** Lit (Geddes 1979)	2014:<br>***Calib:*** NLST, PLCO<br>***Valid:*** PLCO	2014:<br>***Calib:*** NLST, PLCO<br>***Valid:*** PLCO	NA
Goldwasser 2013	2013: TSCE (Meza 2008)<br>***Calib:*** MLP (control arm)	NA	NA	2013:<br>***Calib:*** MLP (control arm)<br>2017:<br>National Center for Health Statistics	2013:<br>CISNET Smoking HR<br>***Calib:*** MLP	2013:<br>Tumor growth (estimated): MLP (control arm)<br>2017:<br>Tumor size: SEER (NR, 1988-2012), NLST (CXR arm)	NA	2013:<br>***Calib:*** MLP (control arm)	NA
Chen 2014	Rice-MD Anderson TSCE Model	NA	NA	Rice-MD Anderson TSCE Model	NCI Smoking History Generator	Tumor growth and metastasis model SEER<br>***Valid:***	NA	Tumor size by stage:<br>***Valid***: SEER	NA

Model Name	Incidence	Prevalence	Mortality (all-cause)	Mortality (DCoD)	Mortality (OCoD	Cancer Progression	Histology Dist.	Stage Dist.	Recur
						SEER (1998-1999)			
UM-LCSs	2014:<br>TSCE (Meza 2008)<br>***Calib:*** NLST, PLCO<br>***Valid:*** PLCO<br>2018:<br>Bach Model (Bach 2013)	NA	NA	2014:<br>SEER<br>***Calib:*** NLST, PLCO<br>***Valid:*** PLCO<br>2019:<br>SEER<br>2022:<br>CISNET Smoking History Generator	2014:<br>***Calib:*** NLST, PLCO<br>***Valid:*** PLCO	NA	2014:<br>***Calib:*** PLCO	2014:<br>***Calib:*** SEER	NA
Hinde 2015	***Calib:*** NDRS Lung Cancer Data Audit (LUCADA)<br>***Valid:*** Expert validation	NA	NA	International Cancer Benchmarkin g Partnership	England Life Tables	Expert elicitation<br>***Calib:*** NDRS Lung Cancer Data Audit (LUCADA)<br>***Valid:*** Expert validation	NA	NA	NA
Shih 2016	NA	NA	NA	Lit (Detterbeck 2008)	NA	NA	NA	Lit (Detterbeck 2008; Fischel 2009)	NA
The Microsimulat ion Lung Cancer (MILC) model	TSCE (Moolgavkar 1990; Heidenreich 1997)<br>***Calib:*** SEER<br>***Valid:*** SEER	NA	NA	National Health Interview Survey; SEER	National Health Interview Survey; SEER	Tumor growth model (Detterbeck 2008); Tumor growth parameters:	NA	NA	NA

Model Name	Incidence	Prevalence	Mortality (all-cause)	Mortality (DCoD)	Mortality (OCoD	Cancer Progression	Histology Dist.	Stage Dist.	Recur
						Lit (Koscielny 1985)			
Treskova 2017	TSCE (Moolgavkar 1990)<br>***Calib:*** Lit (Eberle 2015)<br>***Valid:*** NLST	NA	NA	MIcrosimulation Lung Cancer (MILC) model R Package	Lit (Woloshin 2008), German Life Tables	Lit (McMahon 2012)	Lit (McMahon 2012)	***Calib:*** German UICC Data<br>***Valid:*** NLST	NA
Hofer 2018	***Calib:*** German Centre for Cancer Registry Data	NA	NA	***Calib:*** Lit (Detterbeck 2008)	WHO Mortality Index (Adjusted for LC cases)	***Calib:*** German Centre for Cancer Registry Data	NA	NA	Lit (Pisters 2005)
Exeter NAtural history-Based economic model of Lung cancer screening (ENaBL)	NLST	NLST	NA	England Cancer Registry	England and Wales Life Tables; Continuous Mortality Investigation Mortality Committee; UK Cancer Registry	NLST	NA	NA	NA
Allen 2020	Lit (Meza 2008)	NA	NA	SEER	Lit (Rosenberg 2012)	***Calib:*** MISCAN_Lung Weibull Distributions	NLST	NA	NA
SiMRiSc Lung	integraal kankercentrum Nederland (Netherland Cancer Registry)	NA	Centraal Bureau voor de Statistiek (Statistics Netherlands)	IASLC lung cancer staging project	NA	Tumor growth: I-ELCAP	NA	NA	NA

Model Name	Incidence	Prevalence	Mortality (all-cause)	Mortality (DCoD)	Mortality (OCoD	Cancer Progression	Histology Dist.	Stage Dist.	Recur
Diaz 2021	Lit (Hinde 2015)<br>***Calib:*** Globocan (Men)	NA	NA	EUROCARE 2000<br>***Calib:*** WHO Cancer Mortality Database (Men)	Instituto Nacional de Estadística (Spain National Statisitics Institute)<br>***Calib:*** WHO Cancer Mortality Database (Men)	Lit (Hinde 2015)	NA	NA	NA
Esmaeili 2021	Lit (Yousefi 2021; Yousefi 2019)	NA	NA	Globocan	Iranian life table	Globocan	NA	Pilot Pomeranian lung cancer screening program, Lit (Sobue 2002)	NA
Souliotis 2022	Globocan, Hellenic Statistical Authority	NA	NA	Clinical Expert Opinion	NA	Clinical Expert Opinion	Globocan, Hellenic Statistical Authority	Clinical Expert Opinion	NA
NCC-lung	TSCE (Schultz 2012); Lit (Chen 2015)<br>***Calib:*** China Cancer Registry<br>***Valid:*** China Kadoorie Biobank; NCC nationwide cancer registration	NA	National Mortality Surveillanc e Report	Lit (Goldstraw 2016)<br>***Calib:*** China Cancer Registry<br>***Valid:*** China Kadoorie Biobank; NCC nationwide cancer registration	Smoking history generator	Lit (Chien 2008, Broder 2021)	Lit (Yang 2019)<br>***Calib:*** NCC hospital-based cancer registration	***Calib:*** NCC hospital-based cancer registration	NA

Abbreviations: (DCoD = Disease specific cause of death; OCoD = Other Cause of Death; Calib = Calibration; Valid = Validation; Lit = Literature; FOS-LDS = Framingham Offspring Study Limited Dataset; MLP = Mayo Lung Project; MSKCC LCSP = MSKCC lung cancer screening program; CISNET Smoking HR =

Smoking history and age-dependent annual hazard rates provided by CISNET; CanSPUC = Cancer Screening Program in Urban China; Recur = Recurrence, NCIC CTG Trial = National Cancer Institute of Canada Clinical Trial Group clinical trial)

Table 5. Calibration, validation, and sensitivity analysis of LC NHMs

Model Name	Calibration	Validation	Sensitivity Analysis
Geddes 1979	✗	✓	✗
Manton 1982	✗	✓	✗
MISCAN-Lung	✓	✓	✓
Flehinger 1987	✓	✓	✓
Lung Cancer Policy Model (Population)	✓	✓	✓
Lung Cancer Outcomes Simulator (LCOS)	✓	✓	✓
Longitudinal Multistage Observation Model (FHCRC)	✓	✓	✓
Goldwasser 2013	✓	✓	✓
Chen 2014	✗	✓	✓
UM-LCSs	✓	✓	✓
Hinde 2015	✓	✓	✓
Shih 2016	✗	✗	✓
The Microsimulation Lung Cancer (MILC) model	✓	✓	✗
Treskova 2017	✓	✓	✓
Hofer 2018	✓	✗	✓

Exeter NAtural history-Based economic model of Lung cancer screening (ENaBL)	✗	✗	✓
Allen 2020	✓	✗	✗
SiMRiSc Lung	✗	✗	✓
Diaz 2021	✓	✗	✗
Esmaeili 2021	✗	✗	✓
Souliotis 2022	✗	✗	✗
NCC-lung	✓	✓	✗
